# Supplementary material for: Tryptophan oxidation-induced dynamics highlight why stable γS-crystallin variants aggregate
Source: Biophys Rep (N Y). 2026 Oct 12;6(4):100291. doi: 10.1016/j.bpr.2026.100291 (PMC13722077; doi:10.1016/j.bpr.2026.100291)
Supplement: Supplementary file 1 [file mmc1.pdf]

Biophysical Reports, Volume *6*

Supplemental information

Tryptophan oxidation-induced dynamics highlight why stable γS-crystallin variants aggregate

Yeonseong Seo, Amanda Abiad, Micco Iwase, Tsoler K. Demerdjian, Linyuting Xing, Minseo Kim, Elizabeth M. Wingen, and Rachel W. Martin

Supplemental information for: Tryptophan oxidation-induced dynamics highlight why stable γS-crystallin variants aggregate

Yeonseong Seo[1], Amanda Abiad[1], Micco Iwase[2], Tsoler K. Demerdjian[3], Linyuting Xing[1], Minseo Kim[1], and Rachel W. Martin[1,4,*]

[1]Department of Chemistry, University of California, Irvine, Irvine, CA 92697, USA
[2]School of Biological Sciences, University of California, Irvine, Irvine, CA 92697, USA
[3]School of Population & Public Health, University of California, Irvine, Irvine, CA 92697, USA
[4]Department of Molecular Biology and Biochemistry, University of California, Irvine, Irvine, CA 92697, USA
[*]Correspondence: rwmartin@uci.edu

The following items are available for download online at DOI: 10.17632/xjvy2vw839.1: 1) fluorescence, circular dichroism (CD), and dynamic light scattering (DLS) characterization data for γS-WT, γS-W163(5HTP), and γS-W163S and 2) ^{1}H–^{15}N nuclear magnetic resonance (NMR) relaxation data for γS-WT and γS-W163(5HTP).

Figure S1: γS-W163S sequence was designed with 5′ and 3′ overhangs for Gibson assembly.

```
TGTTTAACTTTAAGAAGGAGATATACCATGGGACATCACCATCACCATCACGAAAATCTTTATTTTCAAGGCAGCAAAACG
GGCACCAAAATCACGTTCTACGAAGACAAAAATTTCCAAGGTCGCCGTTACGACTGTGACTGCGACTGTGCGGACTTCCAC
ACATACCTGTCCCGTTGCAACTCTATCAAAGTTGAAGGCGGCACCTGGGCTGTATACGAACGCCCGAACTTCGCTGGCTAC
ATGTACATCCTTCCGCAGGGCGAATACCCGGAATACCAGCGCTGGATGGGTCTGAACGACCGTCTGTCGTCTTGCCGCGCT
GTACATCTGCCGTCCGGCGGTCAGTACAAAATCCAGATCTTCGAAAAAGGCGACTTCTCCGGCCAGATGTACGAAACCACC
GAAGACTGCCCGTCTATCATGGAACAATTCCACATGCGCGAAATCCACTCTTGTAAAGTTCTGGAAGGCGTATGGATCTTC
TACGAACTGCCGAACTACCGCGGTCGCCAGTACCTGCTGGACAAAAAAGAATACCGCAAACCGATCGACAGCGGCGCGGCC
TCTCCGGCTGTACAGAGCTTCCGTCGTATCGTTGAATAATAACTCGAGCACCACCACCACCACCACTGAGATC
```

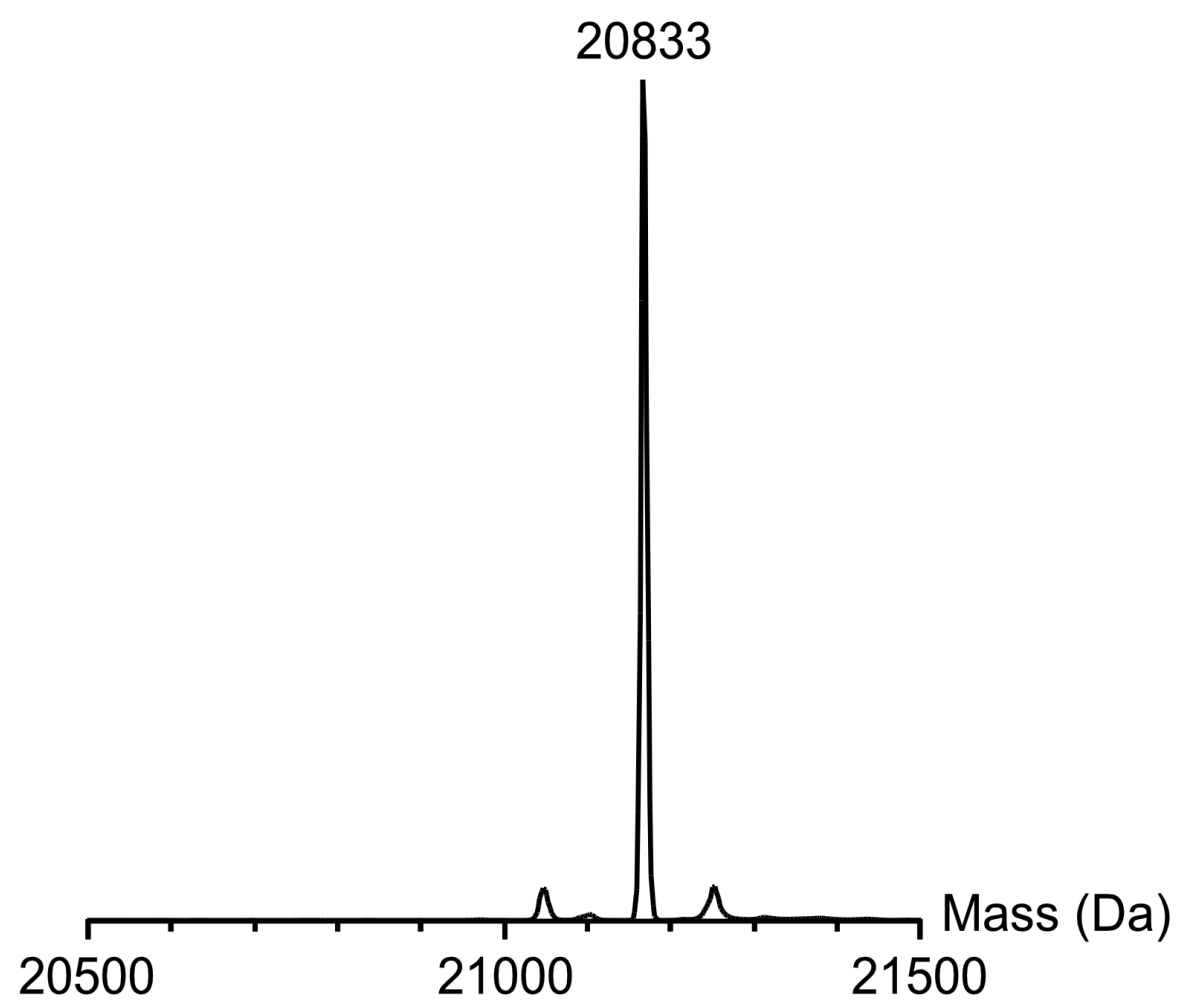

Figure S2: The reconstructed mass spectrum of γS-W163S showed a peak that corresponds to the expected mass calculated from its amino acid sequence.

Table 1: Protein secondary structure analysis of circular dichroism (CD) spectra of γS-WT, γS-W163(5HTP), and γS-W163S suggests a small increase in β-strand content as residue 163 is altered.[1]

**Structure**	**γS-WT**	**γS-W163(5HTP)**	**γS-W163S**
Helix	0.0%	0.0%	0.0%
Strand	49.5%	53.1%	55.7%
Others	50.5%	46.9 %	44.3%

Table 2: Destabilization of γS-W163S was quantified from thermal and chemical unfolding curves and compared to previously published values for γS-WT and γS-W163(5HTP) with permission (license number 6295410703193).[2] Parameters are given as mean $\pm$ standard deviation. Adjusted p-values were calculated using one-way ANOVA with Dunnett's multiple comparison test versus γS-WT.

**Parameter**	**γS-WT**	**γS-W163(5HTP)**	**p-value**	**γS-W163S**	**p-value**
T_m (℃)	78.1$\pm$0.4	72.8 $\pm$0.3	$<$0.0001	66.7 $\pm$0.5	$<$0.0001
Midpoint (M)	2.77 $\pm$0.08	2.47 $\pm$0.02	0.0005	1.73 $\pm$0.03	$<$0.0001
ΔG_{app} (kJ mol^{-1})	6.01 $\pm$0.03	5.5 $\pm$0.8	0.59	9.5 $\pm$0.8	0.0011
m (kJ/mol/M)	$-$0.75 $\pm$0.03	$-$0.7 $\pm$0.1	0.97	$-$1.4 $\pm$0.1	0.0005

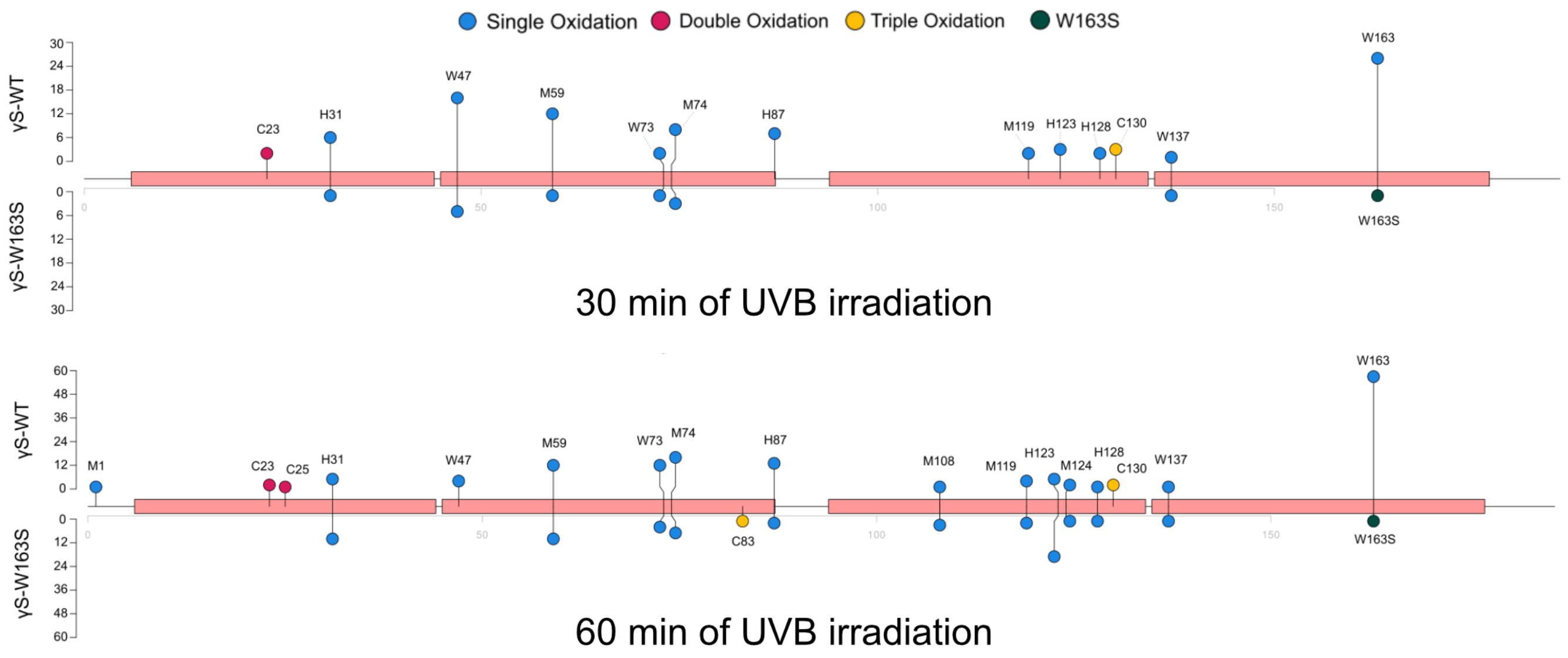

Figure S3: Lollipop plots showing modification sites for γS-WT (up) and γS-W163S (down). The top panel shows modifications after 30 minutes of UV-B irradiation, whereas the bottom panel shows modifications after 60 minutes. Single oxidations are shown in blue, double in red, and triple in yellow. Only the soluble fraction of the protein was digested here: modification sites on insoluble aggregates are therefore not observed.

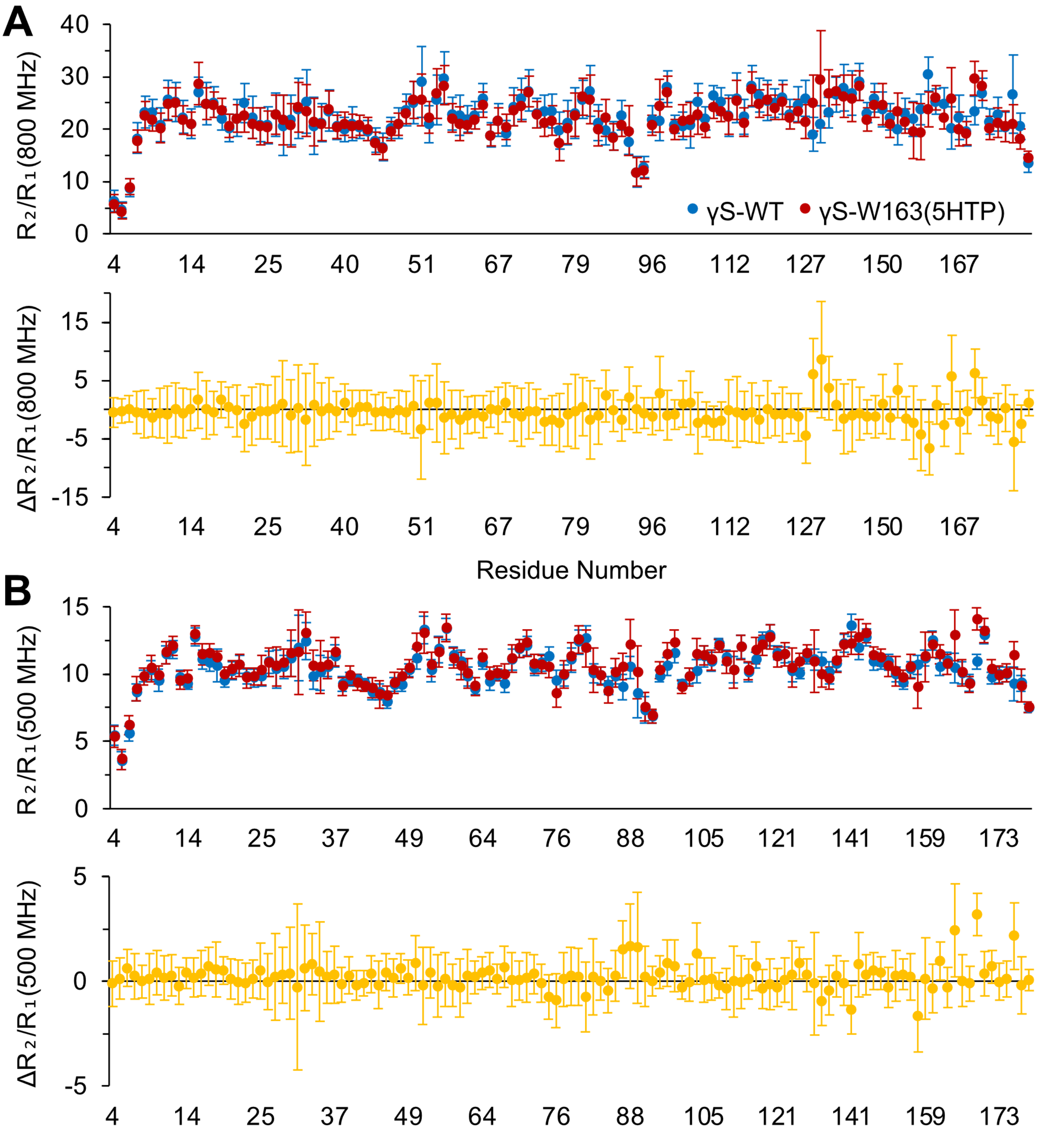

Figure S4: R_2/R_1 of γS-WT and γS-W163(5HTP) were calculated by fitting the ^{1}H–^{15}N NMR relaxation data collected at (A) 800 MHz and (B) 500 MHz. $\Delta R_2/R_1$ are plotted to show perturbations caused by W163 oxidation. Points indicate the calculated value and solid error bars denote the fitting-derived uncertainty (for R_2/R_1) and the propagated uncertainty (for $\Delta R_2/R_1$). Residues that were not detected, assigned, or fitted in either dataset are excluded.

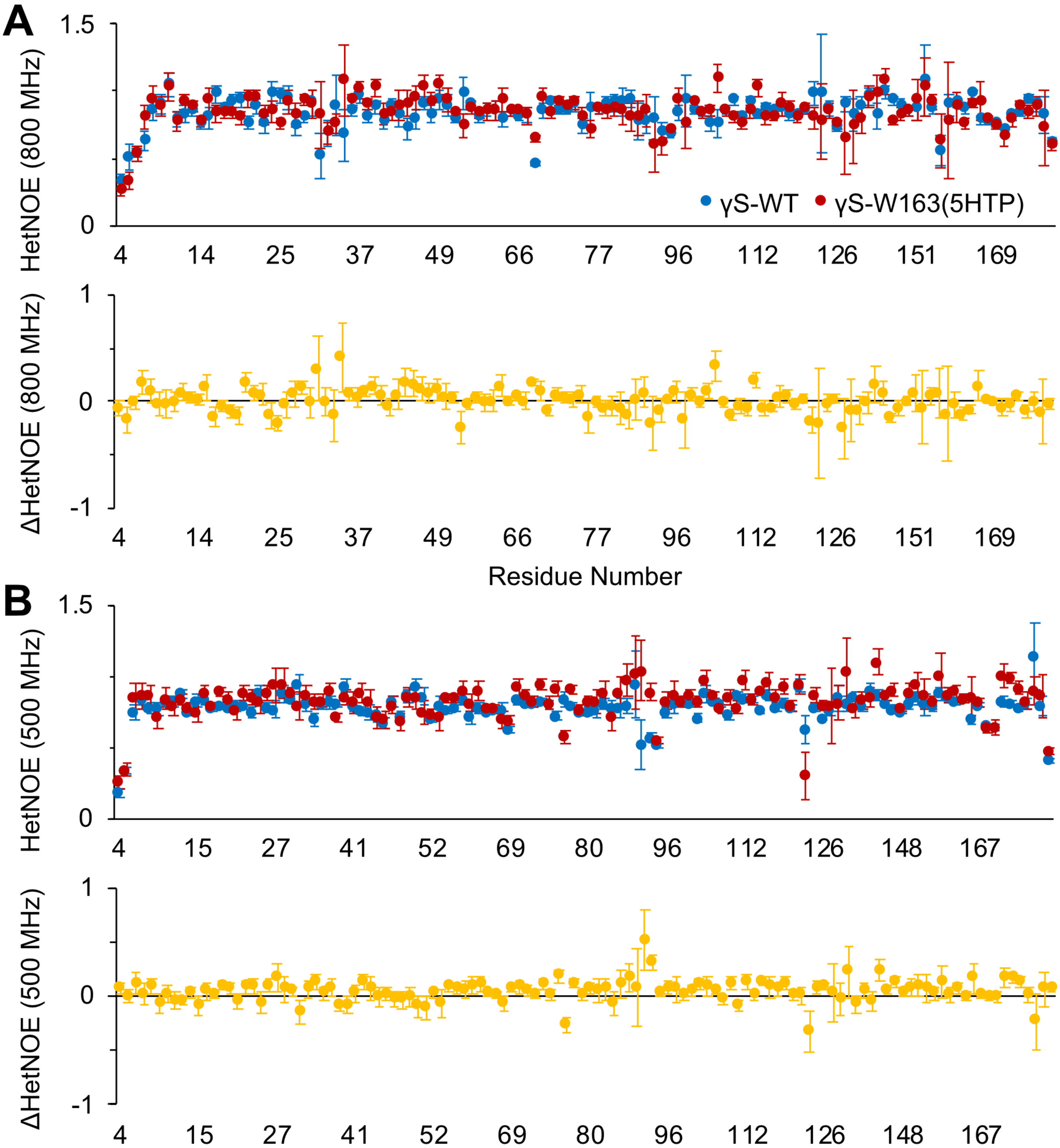

Figure S5: HetNOE of γS-WT and γS-W163(5HTP) were measured at (A) 800 MHz and (B) 500 MHz. ΔHetNOE are plotted to show perturbations caused by W163 oxidation. Points indicate the calculated value and solid error bars denote the intensity-derived uncertainty (for HetNOE) and the propagated uncertainty (for ΔHetNOE). Residues that were not detected, assigned, or fitted in either dataset are excluded.

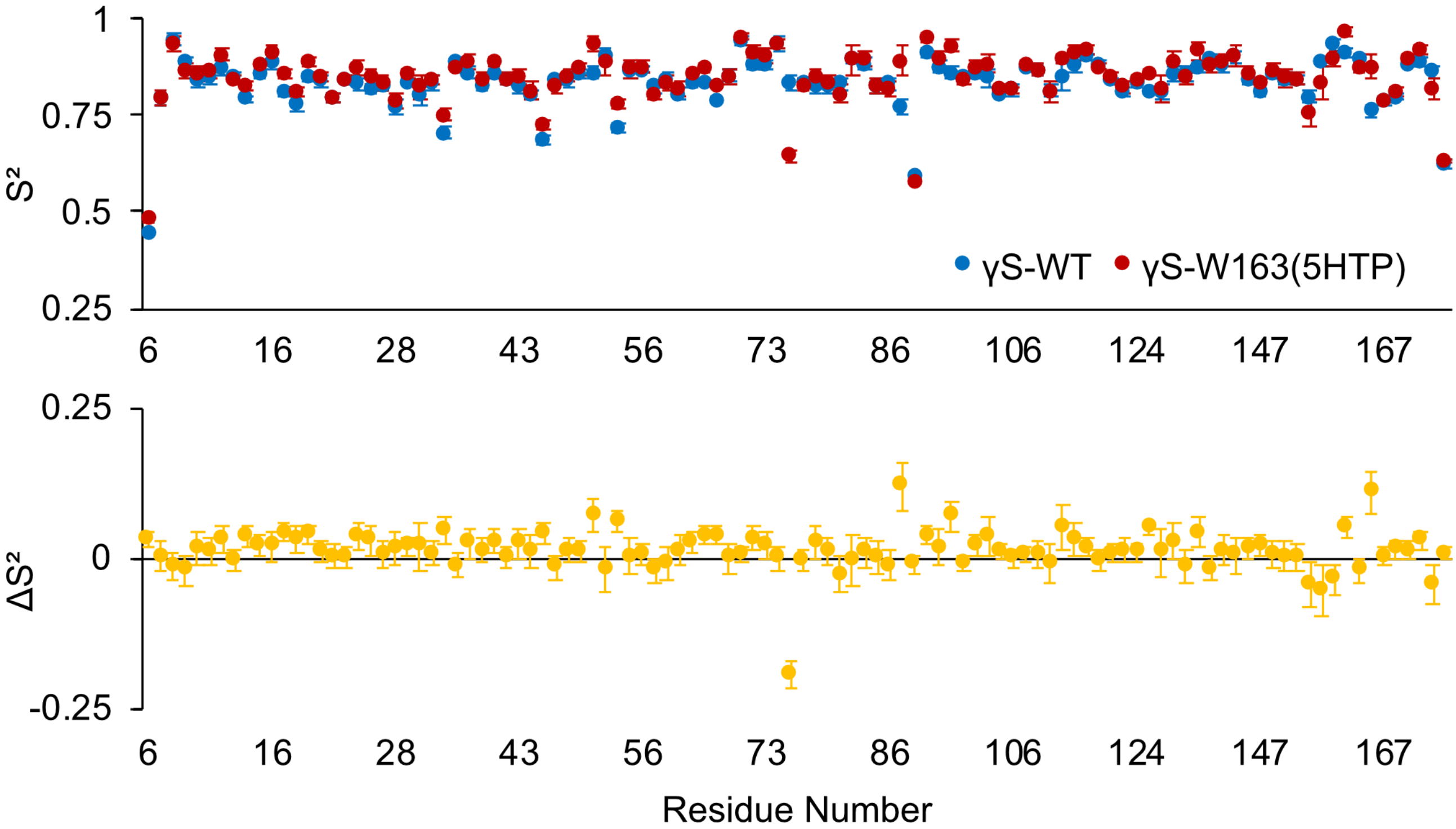

Figure S6: S^2 of γS-WT and γS-W163(5HTP) were estimated via Lipari–Szabo model-free analysis of the ^1H–^{15}N relaxation data. ΔS^2 are plotted to show perturbations caused by W163 oxidation. Points indicate the calculated value and solid error bars denote the fitting-derived uncertainty (for S^2) and the propagated uncertainty (for ΔS^2) of each residue. Residues that were not detected, assigned, or fitted in either dataset were excluded.

References

1. Micsonai, A., Wien, F., Kernya, L., Lee, Y.H., Goto, Y., Réfrégiers, M., and Kardos, J. (2015). Accurate secondary structure prediction and fold recognition for circular dichroism spectroscopy. Proceedings of the National Academy of Sciences *112*, E3095–E3103.

2. Seo, Y., Long, Z.G., Demerdjian, T.K., Avenido, A.A., Butts, C.T., and Martin, R.W. (2026). Mimicking oxidative damage in γS-crystallin with site-specific incorporation of 5-hydroxytryptophan. Biophysical Reports *6*, 100251.
