# Supplementary material for: Tryptophan oxidation-induced dynamics highlight why stable γS-crystallin variants aggregate
Source: Biophys Rep (N Y). 2026 Oct 12;6(4):100291. doi: 10.1016/j.bpr.2026.100291 (PMC13722077; doi:10.1016/j.bpr.2026.100291)
Supplement: Supplementary file 2 [file mmc2.pdf]

Biophysical Reports

ARTICLE

Tryptophan oxidation-induced dynamics highlight why stable γS-crystallin variants aggregate

Yeonseong Seo,[1] Amanda Abiad,[1] Micco Iwase,[2] Tsoler K. Demerdjian,[3] Linyuting Xing,[1] Minseo Kim,[1] Elizabeth M. Wingen,[1] and Rachel W. Martin[1,2,4,*]

[1]Department of Chemistry, University of California, Irvine, Irvine, CA 92697-2025, USA; [2]Department of Molecular Biology and Biochemistry, University of California, Irvine, Irvine, CA 92697-3900, USA; [3]School of Population & Public Health, University of California, Irvine, Irvine, CA 92697, USA; and [4]Department of Ophthalmology and Visual Sciences, University of California, Irvine, Irvine, CA 92697, USA

ABSTRACT Human γS-crystallin (γS) is a highly soluble protein found in the eye lens, where it is densely packed with other proteins to form the refractive tissue required for vision. Mutations in γS can compromise its solubility and cause congenital cataract, while post-translational modifications (PTMs) can lead to age-related cataract. γS is especially susceptible to oxidative PTMs, which accumulate from lifelong exposure to UV light. We previously investigated the effects of side-chain oxidation at tryptophan 163 (W163) by incorporating an oxidation mimic, 5-hydroxytryptophan (5HTP), in a site-specific manner using genetic code expansion. The resulting variant, γS-W163(5HTP), retains the overall fold of the wild-type γS (γS-WT) even under thermal and chemical stress, yet readily forms large oligomers at physiological temperature. Here, we probe the mechanism underlying this behavior. We first characterize γS-W163S, a serine variant in which the indole ring at position 163 is effectively removed, to further define the role of W163 in γS-crystallin structure and stability. We also map changes in backbone dynamics caused by W163 oxidation in γS-W163(5HTP) using solution-state NMR. Together, our findings support the established model in which crystallin aggregation is decoupled from global destabilization: although the oxidative modification at W163 is structurally tolerated through rearrangement of the local hydrogen bond network, the resulting changes in backbone flexibility render aggregation-competent conformations accessible. This work provides mechanistic insights into how covalent damage to crystallin monomers promotes aberrant intermolecular interactions that lead to cataract formation.

WHY IT MATTERS As global populations age, age-related cataract has become a pressing public health concern. Human γS-crystallin (γS) is one of the structural proteins in the eye lens whose aggregation can impair vision by opacifying the tissue. γS is particularly susceptible to oxidative damage induced by daily UV exposure. Such covalent modifications can alter the structure, stability, and aggregation propensity of γS. Oxidation at a core tryptophan residue, W163, was previously found to promote aggregation below physiological temperature. Here, we examine the mechanism of this observation by introducing mutated and chemically modified γS variants as an additional benchmark for how W163 helps keep γS stable and soluble and further characterizing how the internal motions of the protein change due to W163 oxidation.

INTRODUCTION

Biological environments are highly dense, and the macromolecules in these crowded settings frequently collide with one another.[1] Proteins generally populate native conformations in this milieu, carrying out necessary cellular functions via intermolecular interactions. Because proteins continuously sample alternative conformational states, however, transient occupation of higher-energy, non-native conformations can cause aberrant intermolecular contacts.[2,3] The human eye lens is a uniquely crowded system with extremely high concentrations of proteins, reaching over 400 mg mL^{-1}.[4] Such soluble protein systems prevent precipitation by tightly regulating the local concentration via gene expression and protein degradation as well as the physicochemical environment.[5] Virtually no protein turnover takes place in the adult lens, however, leaving conformational dynamics as a key mechanism for limiting exposure to intermolecular interactions.

Submitted May 15, 2026, and accepted for publication October 8, 2026.

*Correspondence: rwmartin@uci.edu

Editor: Rebecca Berlow.

https://doi.org/10.1016/j.bpr.2026.100291

© 2026 The Author(s). Published by Elsevier Inc. on behalf of Biophysical Society.
This is an open access article under the CC BY license (http://creativecommons.org/licenses/by/4.0/).

βγ-Crystallins are the primary structural proteins in the human eye lens; among them is γS-crystallin (γS), which comprises approximately 9% of the total crystallins[6] and is most abundant in the water-rich lens cortex.[7] γS achieves its remarkable solubility through a hydration shell that provides a barrier to direct contact between its monomers.[8] Nevertheless, its solubility can be compromised by factors including mutations, post-translational modifications (PTMs), and environmental stressors. Reduced crystallin solubility can result in aggregation or phase separation that opacifies the lens and causes cataract. For instance, a number of γS variants with a point mutation, such as G18V,[9] V42M,[10] and G57W,[11] are more prone to self-assemble into insoluble aggregates compared to the wild-type γS (γS-WT), providing a mechanistic rationale for their association with congenital cataract. Age-related cataract, on the contrary, is often linked with PTMs: covalent damage, such as deamidation and oxidation of Met, Cys, and Trp, have been reported throughout the sequences of βγ-crystallins isolated from cataractous lenses.[12,13] Namely, deamidations on the surface of γS accumulate and destabilize the folded structure of the protein, resulting in pathological behavior.[14–16]

Catastrophic unfolding is relatively uncommon for βγ-crystallins even in the presence of cataract-associated mutations or PTMs.[17] These globular proteins have tightly packed hydrophobic cores that confer a large thermodynamic bias toward the folded state, which effectively buries their aggregation-prone regions.[18] Pathological interactions arise when the equilibrium between transient conformational states shifts to increase the population and lifetime of locally unfolded yet globally native-like states.[19,20] The P23T mutation in γD, for instance, causes the formation of amorphous aggregates under physiological conditions despite its retention of an overall native-like fold.[21] Intermediates with partial unfolding in the N-terminal domain were also identified along the aggregation pathway of other cataract-prone variants of this protein, such as γD-V75D and γD-W42R.[22] Deamidation also induces conformations that enable non-native intermolecular disulfide bonds between γS monomers, although ensemble-averaged measurements revealed only subtle structural changes.[14,15] Such oxidative damage appears to play a role in promoting crystallin aggregation, but the impact is generally more subtle than large-scale unfolding or even misfolding to form amyloid aggregates.

We were intrigued by the observation of tryptophan 163 (W163) oxidation in γS, both in experimentally damaged protein[23] and naturally aged lens[24] samples. Found in the hydrophobic core of the protein, W163 and three other Trp residues are highly conserved.[7] They not only contribute to the high refractive index[18] but are also essential for dissipating the energy absorbed from UV radiation.[25] In both γD and γS, rapid fluorescence quenching is achieved through energy and charge-transfer mechanisms involving the Trp pairs, neighboring residues, and bound water molecules.[7,26] In a recent study, we reported the preparation and characterization of a γS variant bearing an oxidation mimic, 5-hydroxytryptophan (5HTP), at residue 163.[27] Despite the introduction of a polar hydroxyl group into the hydrophobic core of its C-terminal domain, γS-W163(5HTP) retains the overall fold of γS-WT, with only minor reductions in thermal and chemical stability. Its thermal aggregation propensity, however, is substantially higher, with aggregates beginning to form at physiological temperature. This phenomenon—the lack of direct correlation between protein stability and aggregation propensity—has been observed before, e.g., in β-crystallins.[28] Human βB2 is both thermally and chemically more stable than its bovine ortholog; however, the bovine protein is more resistant to thermal aggregation.[29]

Building on our previous findings, we investigate the role of W163 in reinforcing the robust stability-solubility boundary of γS-crystallin by studying the serine variant (γS-W163S). This mutation effectively removes the indole ring responsible for hydrophobic core packing, thereby eliminating a key stabilizing interaction present in both γS-WT and γS-W163(5HTP). The γS-W163S variant exhibits substantial loss of thermal and chemical stability and increased susceptibility to damage from UV-B radiation, despite only minor structural differences from γS-WT under ambient conditions. Furthermore, we probe how oxidatively modifying W163 alters the internal motions of γS, rendering aggregation-competent states accessible at lower temperatures. We employ solution-state NMR relaxation measurements to characterize the dynamics of γS-W163(5HTP) along its backbone, in comparison to γS-WT. Consistent with the modest effects of this single oxidation on the structure and stability of γS, we find that γS-W163(5HTP) has backbone dynamics similar to those of γS-WT overall. Localized perturbations in rigidity that counteract one another are identified, particularly around the site of mimicked PTM. Taken together, our results illustrate how W163 is integral to maintaining the stability and solubility of γS by supporting a network of intra- and intermolecular interactions that biases the conformational ensemble toward native-like states.

MATERIALS AND METHODS

Expression vector preparation

Invitrogen *E. coli* DH5α cells were purchased from Thermo Fisher (Waltham, MA) and used for plasmid

preparation. The pET28a(+) vectors containing the genes for γS-WT and γS-W163(5HTP), along with an N-terminal hexahistidine tag and a tobacco etch virus (TEV) protease cleavage site, were previously purchased from Twist Bioscience (South San Francisco, CA).[16,27] The QIAprep spin miniprep kit was purchased from Qiagen (Germantown, MD) and used for plasmid purification. The gene fragment for His-tagged γS-W163S (Figure S1) was purchased from Twist Bioscience. Restriction enzymes NcoI and XhoI and Gibson assembly master mix were purchased from New England Biolabs (Ipswich, MA) and used to assemble the pET28a(+) vector containing the gene for γS-W163S. Genes of interest within plasmid samples were verified by Sanger sequencing using the T7 universal primer provided by GENEWIZ from Azenta (South Plainfield, NJ).

Protein expression

Novagen *E. coli* Rosetta (DE3) cells were purchased from Thermo Fisher and used to express γS-WT and γS-W163S (Addgene #255529) using protocols adapted from previous reports.[27,30] Chemically competent cells were transformed with the corresponding expression vectors. Single colonies were picked and grown in Luria-Bertani (LB) broth (1% tryptone, 0.5% yeast extract, and 1% sodium chloride in water) with 50 μg mL^{-1} kanamycin and 20 μg mL^{-1} chloramphenicol overnight at 37°C with shaking. The overnight cultures were diluted 1:20 into 500 mL of LB broth supplemented with the antibiotics and 1 mM magnesium sulfate. The cells were allowed to grow at 37°C until an optical density at 600 nm (OD_{600}) of 0.8–1.0 was reached, then overexpression was induced by adding a final concentration of 1 mM IPTG and incubating for 16–18 h at 25°C with shaking.

ATMW-BL21 cells containing the pEVOL suppression plasmid with *glnS* and *lpp* promoters (pEVOL-*glnS*-*lpp*) were gifted by the Chatterjee laboratory[31] and used to express γS-W163(5HTP) as previously described.[27] Chemically competent cells were transformed with the γS-W163(5HTP) expression vector (Addgene #255530). A single colony was picked and grown in LB broth with 50 μg mL^{-1} kanamycin, 20 μg mL^{-1} chloramphenicol, 95 μg mL^{-1} spectinomycin, 15 μg mL^{-1} Zeocin, and 10 μg mL^{-1} gentamicin overnight at 37°C with shaking. The overnight culture was diluted 1:20 into 500 mL of LB broth supplemented with the antibiotics and final concentrations of 1 mM magnesium sulfate and 4 mM 5HTP. The cells were allowed to grow at 37°C until an OD_{600} of 0.8–1.0 was reached, then overexpression was induced by adding a final concentration of 1 mM IPTG and incubating for 20–24 h at 25°C with shaking. The expression time was lengthened relative to our standard practice in order to compensate for the slower growth of ATMW-BL21 strain containing the suppressor system.

^{15}N-labeled samples of γS-WT, γS-W163(5HTP), and γS-W163S were expressed in the bacterial strains specified above using protocols adapted from previous reports.[27,32] Overnight cultures grown from single colonies were diluted 1:20 into 500 mL of LB broth supplemented with the corresponding antibiotics. The cells were allowed to grow at 37°C until an OD_{600} of 1.0 before centrifugation at 5,000 rpm at 4°C for 20 min. The pellets were resuspended in 500 mL of ^{15}N-labeled M9 minimal medium (48 mM sodium phosphate, 22 mM potassium phosphate, 9 mM sodium chloride, 2 mM magnesium sulfate, 0.1 mM calcium chloride, 18 mM ^{15}N-labeled ammonium chloride, and 55 mM glucose in water [pH 7.0]) supplemented with the corresponding antibiotics and 5HTP as necessary. The labeled cultures were incubated at 37°C for 1 h, then overexpression was induced by adding a final concentration of 1 mM IPTG and incubating for 40–44 h at 18°C with shaking.

Protein purification

Corning disposable sterile bottle-top filters with a 0.45 μm pore size were purchased from Fisher Scientific (Pittsburgh, PA) and used to sterilize purification buffers (0–500 mM imidazole, 50 mM sodium phosphate, 300 mM sodium chloride, and 0.05% sodium azide in water [pH 7.0]). Cells from the expression cultures were harvested by centrifugation at 4,000 rpm at 4°C for 30 min, flash-frozen in liquid nitrogen, and stored at −80°C. A frozen pellet was resuspended in 10 mL of lysis buffer (25 mM imidazole) per gram and supplemented with 1 mg mL^{-1} lysozyme, 0.1 mg mL^{-1} deoxyribonuclease I, 0.7 μL mL^{-1} β-mercaptoethanol, and a final concentration of 10 mM magnesium sulfate. The resulting suspension was lysed by sonication (2 s on/off, 15 min, 40% amplitude) and clarified by centrifugation at 13,000 rpm at 4°C for 45 min. Fisherbrand sterile syringe filters with 0.22-μm PVDF membranes were purchased from Fisher Scientific and used to further clarify the supernatant.

HisTrap HP (Ni-IMAC) and HiLoad Superdex 75-pg (size-exclusion chromatography [SEC]) columns were purchased from Cytiva (Marlborough, MA). The supernatant was first loaded onto an Ni-IMAC column, and a gradient of 25–500 mM imidazole was used to elute the His-tagged protein. Fractions were collected and analyzed using a NanoDrop One spectrophotometer for A_{280} to approximate the concentration with the extinction coefficient for γS-WT, 41,040 M^{-1}

cm^{-1}.[33] 1 mg of Loening's TEV Hexa[34] (prepared in-house) was added per 150 mg of protein to cleave the His tag. Spectra/Por dialysis tubing (6–8 kDa) was purchased from Repligen (Waltham, MA) and used to dialyze the resulting mixture in dialysis buffer (50 mM sodium phosphate and 150 mM sodium chloride in water [pH 7.0]) at 4°C for 16 h with stirring. Reapplication to the Ni-IMAC column removed the TEV, and the collected fractions were reduced with a final concentration of 1 mM dithiothreitol (DTT) before they were loaded onto an SEC column. The cleaved protein was eluted using SEC buffer (0 mM imidazole) and dialyzed in water. Amicon ultracentrifugal filters (3 kDa) were purchased from MilliporeSigma (Burlington, MA) and used to concentrate the protein before aliquoting and lyophilizing for storage at −80°C.

Mass spectrometry

Purified protein samples were analyzed by liquid chromatography-mass spectrometry (LC-MS) (Xevo G2-XS QTof) equipped with a phenyl column (ACQUITY UPLC BEH VanGuard) from Waters (Milford, MA). Intact proteins were separated from buffer salts using a gradient of 0%–90% buffer B (acetonitrile) in buffer A (0.1% formic acid in water) at a 0.2 mL min^{-1} flow rate for 5 min. Electrospray ionization (ESI) was run in positive mode across 400–4,000 Da with a capillary voltage of 3,000 V and a cone voltage of 40 V, with nitrogen as desolvation gas at 350°C and a total flow of 800 L h^{-1}. Mass spectra were reconstructed from the charge state series using the MaxEnt1 algorithm from the MassLynx software provided by Waters, as seen in Figure S2.

Biophysical characterization

Lyophilized protein samples were reconstituted in phosphate buffer (10 mM sodium phosphate in water, pH 7.0) that was degassed under vacuum and sterilized by filtration. The samples were reduced by adding a final concentration of 1 mM DTT and incubating on ice for 1 h. Samples were filtered using Whatman Anotop syringe filters with a 0.02-μm membrane from Cytiva. Protein concentrations were approximated using the extinction coefficient for γS-WT, and dilutions were performed with phosphate buffer, unless specified otherwise.

A Jasco J-810 spectropolarimeter was used to measure molar ellipticity of circularly polarized light of protein samples (0.1 mg mL^{-1}). The final circular dichroism (CD) spectra between 190 and 260 nm were acquired at ambient temperature. Each spectrum was analyzed for protein secondary structure using BeStSel software.[35] Molar ellipticity at 218 nm as a function of temperature was used to probe thermal unfolding of concentrated samples (0.25 mg mL^{-1}, 10 mM sodium phosphate, and 150 mM sodium chloride in water [pH 7.0]) as previously described.[27,36] The molar ellipticity was normalized to the fraction of unfolded protein by setting the lowest value as fully folded and the highest value as fully unfolded. Midpoint temperatures (T_m) were calculated by fitting the data to a nonlinear regression model using the 4-parameter logistic (4PL) curve in the Prism software provided by GraphPad (San Diego, CA). This model is commonly used to fit two-state unfolding data to a sigmoidal curve defined by a midpoint, slope, and lower and upper plateaux.[37]

A Cary 60 UV-visible spectrophotometer and a Cary Eclipse fluorimeter were used to measure absorbance and fluorescence of protein samples (1 mg mL^{-1}) at ambient temperature. Emission spectra were first collected using the absorbance λ_{max} as the excitation wavelength. The resulting emission λ_{max} was then used to scan for the optimal excitation wavelength, which was subsequently used to acquire the final emission spectra between 300 and 500 nm. A TECAN Spark multimode microplate reader was used to monitor changes in emission as a function of guanidine hydrochloride (Gdn-HCl) concentration to probe chemical unfolding as previously described.[27,36] Dilute samples (0.1 mg mL^{-1}, 0–6 M Gdn-HCl in 0.2-M increments, 10 mM sodium phosphate, and 50 mM sodium chloride in water [pH 7.0]) were incubated at ambient temperature for 48 h with shaking and then excited at 260 nm to record emission spectra between 300 and 400 nm. The fluorescence intensity ratio at 355–325 nm was normalized to the fraction of unfolded protein by setting the lowest value as fully folded and the highest value as fully unfolded. Thermodynamic parameters were calculated assuming two-state unfolding as previously described.[38]

A Malvern Zetasizer Nano-ZS was used to monitor aggregation of protein samples (1 mg mL^{-1}) as a function of temperature. The average particle size was determined from dynamic light scattering (DLS) correlograms measured over a temperature range of 25°C–80°C in 1°C increments.

UV irradiation and characterization of irradiated protein

Recombinant γS-WT and γS-W163S were prepared at a final concentration of 0.981 and 0.967 mg/mL, respectively, measured with a NanoDrop One spectrophotometer (ThermoScientific) in 20 mM potassium phosphate monobasic (Sigma-Aldrich, ACS grade, ≥99.0%) and 100 mM potassium chloride (Fisher

Chemical, ACS grade, ≥99.0%) in Nanopure water (pH 7.60) buffer. For each experimental condition, 200 μL of protein solution was transferred to two 200-μL cuvettes (DiluCell). Samples were irradiated using two fluorescent UV-B broad-band bulbs (UVP F15T8) positioned approximately 5 inches (~12.5 cm) from each sample. The optical power at this distance was measured as 20 mW using a Thorlabs power meter. Samples were irradiated for 0, 30, 45, and 60 min in duplicate, with separate samples used for each irradiation time. The unirradiated samples (0 min) served as controls. Following irradiation, samples were immediately assessed for turbidity by measuring absorbance at 600 nm (A_{600}) using the NanoDrop One. Measurements were performed using 2 μL of sample. Samples were then processed for bottom-up proteomic analysis using a reduction, alkylation, and tryptic digestion workflow. Dithiothreitol was added to a final concentration of 47.6 mM, and samples were incubated at 80°C for 10 min. Samples were then alkylated with acrylamide for a final concentration of 87.0 mM and incubated at 80°C for 10 min. DTT was added to quench excess acrylamide for a final concentration of 115.4 mM and incubated at 80°C for 10 min. Trypsin Gold (Promega, MS grade) was added to each sample with a final concentration of 3.70 ng μL^{-1} and incubated overnight at 37°C. Following digestion, samples were acidified with 20 μL of 1% formic acid (Fisher Chemical, ACS grade, ≥88.0%) in water, centrifuged at 14,100 × *g*, and prepared for LC-MS/MS analysis. UV irradiation, turbidity measurements, and sample preparation were performed independently for each replicate.

Samples (control or UV-treated) were precipitated using a 10× dilution of acetonitrile, followed by centrifugation and removal of the supernatant. Pellets were resuspended in 200 μL of 50 mM ammonium bicarbonate (pH 8) buffer, followed by the addition of 1 μL of 1 M DTT (in H_2O). The samples were heated at 95°C for 15 min, followed by the addition of 5 μL of 1 M acrylamide (in water), and again incubated at 95°C for 15 min. After incubation, the samples were again diluted 10× with acetonitrile, centrifuged, and the supernatant removed. The samples were resuspended in 200 μL of ammonium bicarbonate buffer, followed by the addition of MS grade trypsin (1 μg in 10 μL of 50 mM acetic acid) and 1 μL of 1 M DTT. Samples were then incubated overnight at 37°C and centrifuged, and the supernatant was loaded into LC-MS vials for analysis.

The samples were analyzed on a Thermo QExactive LC-MS spectrometer with a HESI source, using a Waters Phenyl Hexyl column, with mobile phases A (0.1% formic acid in H_2O) and B (0.1% formic acid in acetonitrile) on a 60-min gradient (0.4 mL min^{-1}) as follows: (1) 1-min equilibration at 0% B prior to injection, (2) 2-min isocratic A, (3) 47-min linear ramp to 25% B, (4) 5-min linear ramp to 100% B, (5) 3-min isocratic B, (6) 1-min ramp down to 0% B, and (7) 2-min equilibration back to 0% B with flow reduced to 0.05 mL/min at the last 0.1 min. The source had sheath gas set to 60, auxiliary gas at 30, no sweep gas, spray voltage 3.5 kV, capillary temp at 300°C, S-lens RF level at 50, and auxiliary gas heated to 350°C.

The MS experiment was a Top9 DDA experiment, using lockmass correction to known background ions. The default charge state was assumed to be 1. The cycle includes an MS1 scan at 35k resolution, AGC target 3E6, maximum IT of 50 ms, and scan range 150–2,000 Da. The MS2 scans were each 17.5k resolution, AGC target 5E5, maximum IT of 50 MS, with a loop count of 9. The isolation window was 3 *m/z* with offset +0.5 *m/z*. Stepped NCE of 20–30–40 was used. Minimum AGC target for MS2 was 1E3, with apex trigger of 2–8 s and charge exclusion of unassigned or 1. Peptide matching was off, exclude isotopes was on, and a dynamic exclusion of 3 s was on.

Peptide and PTM data were processed using the db.peptides export from PEAKS (Bioinformatics Solutions). The number of features reported for each experimental condition was used to map PTMs to their corresponding sites on the protein sequences. The total number of features for each condition was used as the total peptide count. Oxidative PTMs with an AScore of greater than 20 were included in the PTM analysis and included in the PTM summary table(s) and lollipop plots using M2Vis.[39]

NMR

Lyophilized ^{15}N-labeled protein samples were reconstituted in phosphate buffer (10 mM sodium phosphate in water [pH 7.0]) that was degassed under vacuum and sterilized by filtration. For ^{1}H-^{15}N heteronuclear single-quantum coherence (HSQC; hsqcfpf3gpphwg), ^{15}N-labeled protein samples (final concentration of 0.5 mM; 10 mM sodium phosphate in water supplemented with 10% deuterium oxide and 0.5 mM sodium trimethylsilyl propanesulfonate [DSS; pH 7.0]) were used, and the experiments were performed on a Bruker Avance Neo 800 MHz. ^{1}H shifts were referenced to the internal standard (DSS), and ^{15}N shifts were referenced indirectly. The spectrum of γS-W163S was referenced to that of γS-WT to offset a small unintended temperature difference. The spectra were processed with the TopSpin software by Bruker and the CcpNmr Analysis V3 software provided by CCPN.[40] For relaxation (T_1, T_2, and

heteronuclear NOE [HetNOE]) experiments, the samples (1 mM) were prepared by buffer exchange (20 mM sodium phosphate in water [pH 7.0]) via centrifugation and supplemented with final concentrations of 10% deuterium oxide and 0.5 mM DSS. Relaxation experiments were performed on Avance Neo 800 MHz and 500 MHz spectrometers using the hsqc1etf3gpsi3d, hsqc2etf3gpsi3d, and hsqcneof3gpsi3d pulse programs provided by Bruker (Billerica, MA). ^{15}N longitudinal relaxation rates (R_1) were measured using an inversion recovery pulse sequence with relaxation delays of 20, 60, 200, 400, 800, 1,600, and 2,400 ms. ^{15}N transverse relaxation rates (R_2) were measured using a Carr-Purcell-Meiboom-Gill (CPMG) pulse sequence with relaxation delays of 15.7, 31.4, 62.8, 125.6, 157, 188.4, 219.8, and 251.2 ms. Delays of 60 and 1,600 ms for R_1 and 31.4 and 188.4 ms for R_2 were acquired in triplicate. Steady-state ^{1}H-^{15}N HetNOE spectra were acquired with and without an initial proton saturation of 5 s. Phase correction and spectral referencing (^{1}H shifts directly to DSS and ^{15}N shifts indirectly) were performed using the TopSpin software provided by Bruker. R_1, R_2, and HetNOE calculations were performed using CcpNmr Analysis V3 software. The resulting relaxation data were analyzed to obtain dynamic parameters, including average generalized order parameters (S^2) and the overall rotational correlation times (τ_m), using the FAST-Modelfree software[41] provided through the NMRbox platform.[42]

RESULTS

The W163S mutation is minimally perturbative under ambient conditions

Disrupting the tryptophan clusters within γ-crystallins via covalent damage is thought to have structural consequences. Molecular dynamics (MD) simulations of Trp photo-oxidation in bovine γB-[43] and human γD-crystallins[44] showed that such PTMs allow solvent penetration into their hydrophobic interiors, which accelerates unfolding and facilitates aggregation via covalent crosslinking. Based on our characterization of γS-W163(5HTP), W163 oxidation is well tolerated, with the modified protein largely retaining the native fold and stability of γS-WT. γS-W163(5HTP) is nevertheless more aggregation prone, particularly under thermal stress.[27] We sought to further distinguish the effects of adding a polar hydroxyl group to the C-terminal hydrophobic core from those of the aromatic indole ring of W163. We specifically chose the serine variant (γS-W163S) because the corresponding missense mutation is reported in human genomic data.[45] Although the clinical significance of this mutation is so far unknown, we hypothesized that this mutation would effectively remove the thermodynamic bias toward folding and the photoprotection against UV damage provided by the indole chromophore.

The overall fold of γS-W163S was compared to that of γS-WT and γS-W163(5HTP) using CD spectroscopy, which detects the absorption of circularly polarized light to reveal protein secondary structure (Figure 1A). The CD spectrum of γS-W163S shows a strong negative signal at 218 nm, indicative of intact β-sheet secondary structure (secondary structure percentages are tabulated in Table S1),[46] and its spectrum resembles that of γS-WT. This suggests that the Greek key motifs in γS-W163S remain substantially intact despite the loss of a core nonpolar moiety. The hydrodynamic radii of these γS variants, estimated from DLS measurements at 25°C, are as follows: γS-WT, 4.85 $\pm$ 0.03 nm; γS-W163(5HTP), 5.00 $\pm$ 0.09 nm; and γS-W163S, 4.6 $\pm$ 0.2 nm (the values are reported as the mean $\pm$ one standard deviation [$n = 3$]). These values are slightly larger than the previously reported radius of a γS monomer (between 2.6 and 3.4 nm),[47] perhaps due to a small population of dimers, as are sometimes observed in aged samples. Nevertheless, the average particle size of γS-W163S is comparable to that of γS-WT when measured under the same conditions, suggesting that the slight unfolding observed with γS-W163(5HTP) is absent in γS-W163S.

The intrinsic Trp fluorescence is often employed as a sensitive probe to monitor the conformational changes in proteins, capable of detecting any increases in local hydration that manifest as quenching and red-shifting of the emission.[48] It is particularly useful in characterizing the folding status of γ-crystallins, given their conserved Trp pairs that engage in Förster resonance energy transfer (FRET) within each domain.[7,25] As previously reported, the emission wavelength and intensity undergo slight changes when W163, the C-terminal FRET acceptor, is substituted with 5HTP in γS-W163(5HTP).[27] On the contrary, the λ_{em} of γS-W163S (327 nm) is even closer to that of γS-WT (328 nm). Importantly, an increase of over 2.5-fold in intensity is observed in the absence of a chromophore at residue 163: fluorescence emission at their respective λ_{em} values is 121 arbitrary units (a.u.) for γS-WT compared to 307 a.u. for γS-W163S. The effectiveness of energy dissipation via FRET in γS-WT is further highlighted when considering that γS-W163S has one fewer chromophore that absorbs and emits at these wavelengths. This trend was also observed by Chen et al. using the γS-W163F variant, which exhibits a 1.5-fold increase in fluorescence intensity relative to γS-WT, likely due to the weak quenching effect of phenylalanine.[7]

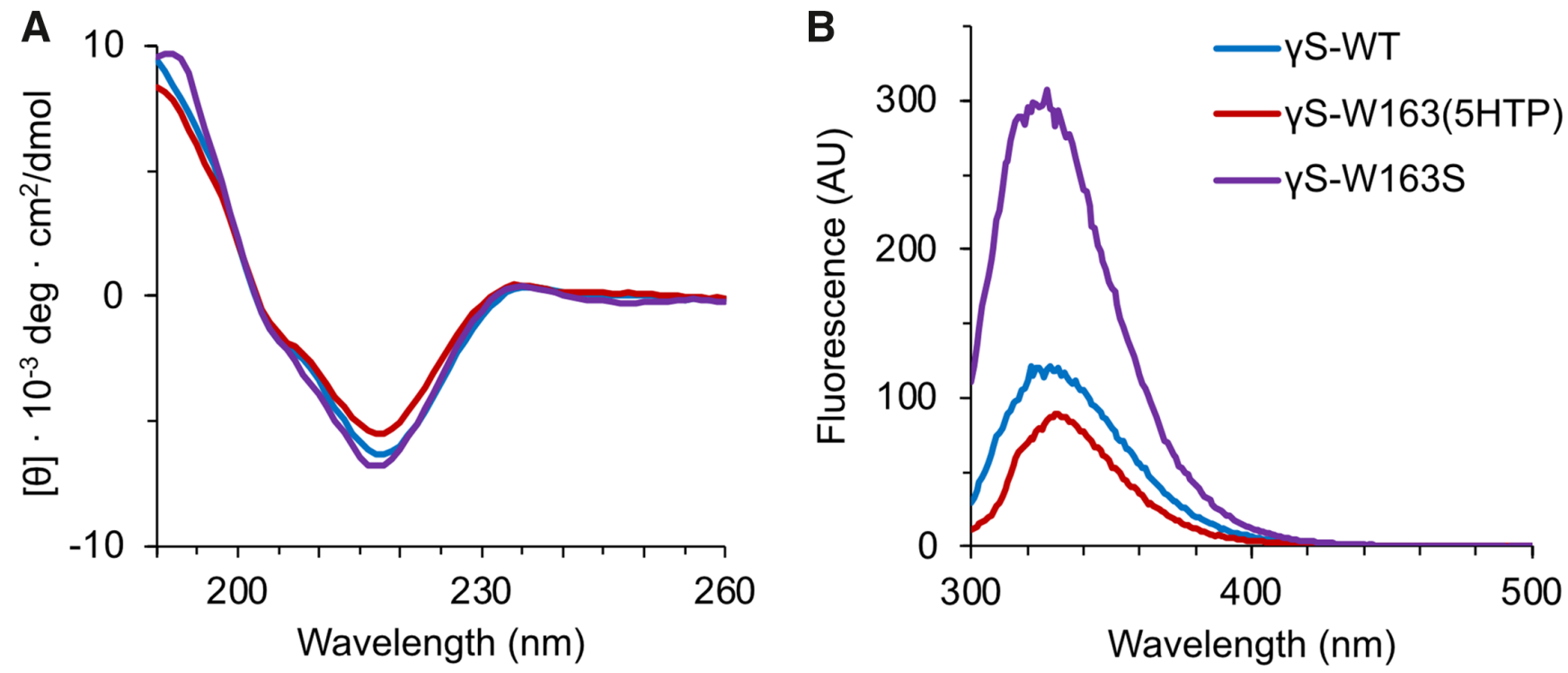

Figure 1. γS-W163S retains the overall structure of γS-WT at ambient temperature. Results for γS-WT and γS-W163(5HTP) were previously published[27] and are replotted with permission (license number 6295410703193) for purposes of comparison. (A) The CD spectrum of γS-W163S largely resembles that of γS-WT, indicating the retention of β-sheet packing despite the mutation. (B) The intrinsic Trp fluorescence of γS-W163S is much higher in intensity than that of γS-WT and γS-W163(5HTP), due to the loss of the C-terminal FRET acceptor.

γS-W163S is notably less stable than γS-WT and γS-W163(5HTP)

The loss of β-sheet structure within γS-W163S as a function of temperature was monitored using CD spectroscopy and compared to that of γS-WT and γS-W163(5HTP) (Figure 2A). Its midpoint unfolding temperature (T_m) is 66.7°C, about 11.4°C lower than that of γS-WT—an additional 6.1°C reduction from that of γS-W163(5HTP) (Figure 2B; Table S2). Such thermal instability is comparable to that of γS-G18V (T_m = 63.6°C), a variant associated with congenital cataract.[17] Surprisingly, this compromise in stability does not lead to premature thermal aggregation as previously seen with γS-W163(5HTP).[27] The apparent particle size of γS-W163S, measured using DLS, remains consistent with a monomer at physiological temperature and starts to form larger aggregates around 43°C, which matches the T_{agg} of γS-WT (Figure 2C). The aggregation profile of γS-W163S is steeper, though, surpassing the reliable size range for the instrument (>500 nm) at 49°C, compared to 54°C for γS-WT.

γS-W163S was also subjected to chemical denaturation, which was quantified using the red shift of its fluorescence as a function of Gdn-HCl concentration (Figure 3A). Similar to γS-WT and γS-W163(5HTP), γS-W163S exhibits a largely sigmoidal unfolding curve, suggesting a two-state mechanism. Under this assumption, the ΔG of denaturation (ΔG_{app}) was calculated using the ratio of unfolded to folded conformations at each equilibrium (Figure 3C).[38,49] The midpoint denaturant concentration shows a similar trend to that of the T_m: half of the γS-W163S ensemble is denatured at 1.73 M Gdn-HCl, which is about 1 M lower than the midpoint concentration of γS-WT and

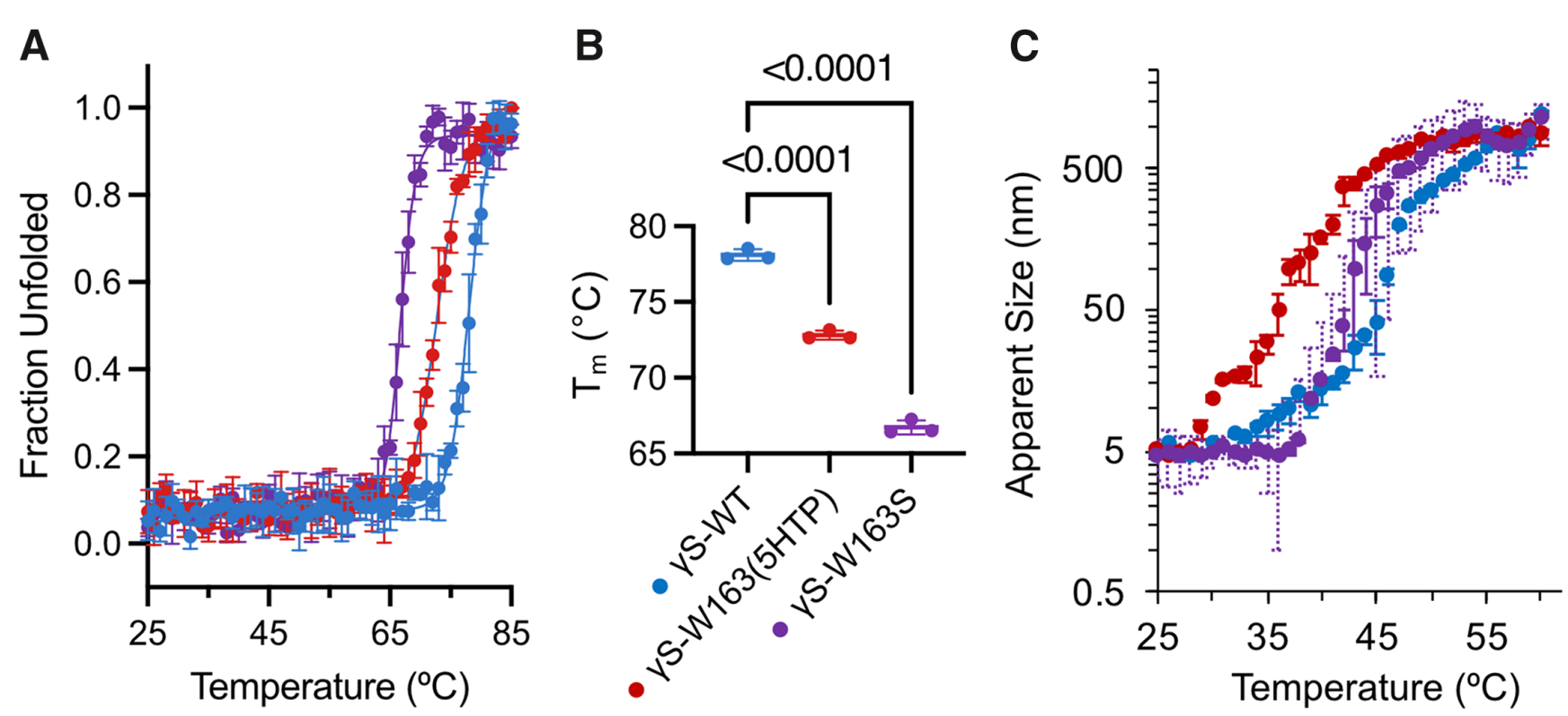

Figure 2. γS-W163S exhibits compromised thermal stability. Results for γS-WT and γS-W163(5HTP) were previously published[27] and are replotted with permission (license number 6295410703193) for purposes of comparison. (A) Thermal unfolding monitored by molar ellipticity at 218 nm demonstrates that γS-W163S exhibits reduced thermal stability compared to γS-WT and γS-W163(5HTP). Points indicate mean values, solid error bars denote standard deviations, and solid lines show unfolding curves. (B) Midpoint unfolding temperature values (n = 3) are shown as points with their mean and standard deviation denoted by solid bars. (C) Thermal aggregation detected by DLS indicates that γS-W163S retains thermal aggregation propensity similar to that of γS-WT. Representative data are plotted for γS-W163S with 95% confidence intervals calculated from three sample replicates shown as dotted error bars.

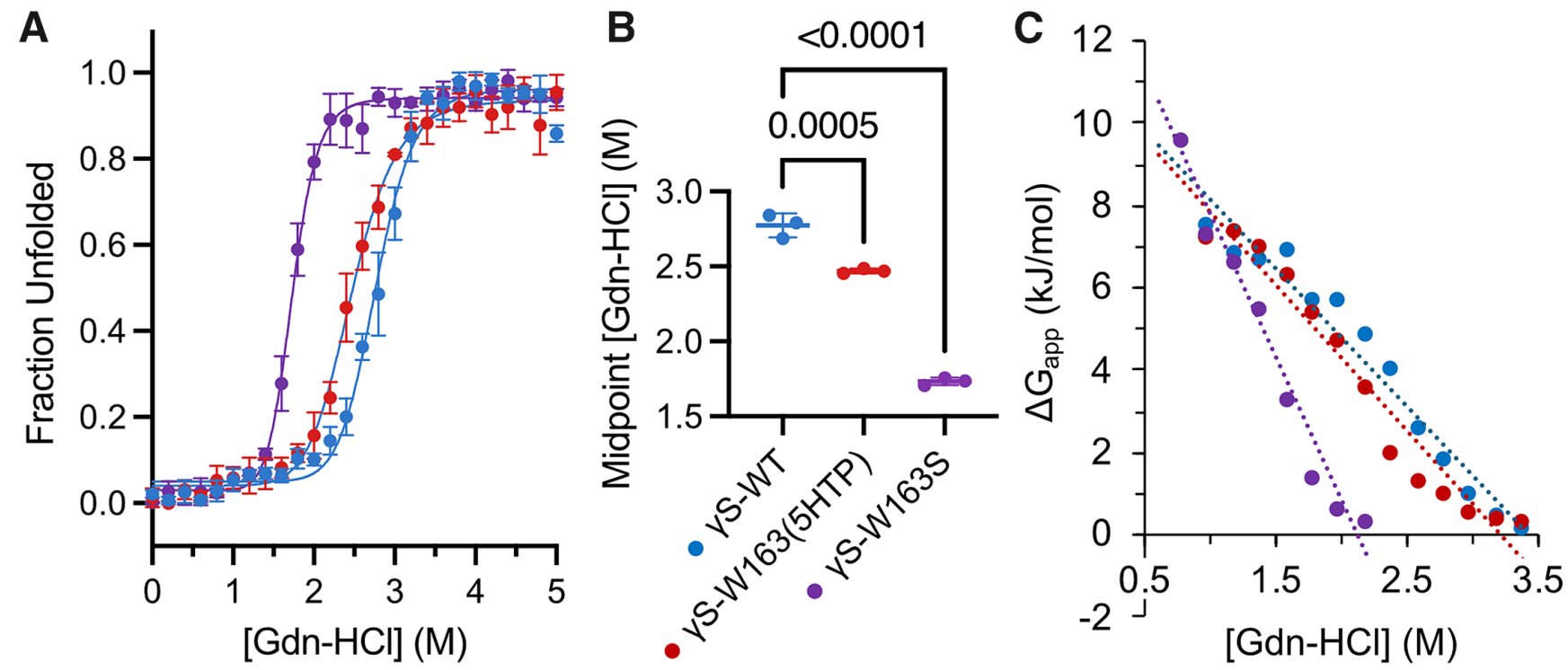

Figure 3. γS-W163S exhibits reduced chemical stability. Results for γS-WT and γS-W163(5HTP) were previously published[27] and are replotted with permission (license number 6295410703193) for purposes of comparison. (A) Chemical unfolding monitored by Trp fluorescence shows that γS-W163S exhibits reduced chemical stability compared to γS-WT and γS-W163(5HTP). Points indicate mean values, solid error bars denote standard deviations, and solid lines show unfolding curves. (B) Midpoint denaturant concentration values ($n = 3$) are shown as points with their mean and standard deviation denoted by solid bars. (C) Linear relationships between ΔG_{app} and [Gdn-HCl] can be drawn for γS-W163S assuming a two-state unfolding mechanism, using $K = e^{-\Delta G_{app}/RT}$.[38] Points indicate mean values, and dotted lines indicate linear relationships.

0.74 M lower than that of γS-W163(5HTP) (Figure 3B). Furthermore, the |*m*|-value of γS-W163S is nearly doubled ($m = -1.4$) compared to those of γS-WT ($m = -0.75$) or γS-W163(5HTP) ($m = -0.7$) which can be interpreted as a larger change in solvent-accessible surface area upon unfolding (Table S2).[50]

To further investigate the susceptibility of γS-W163S to photodamage, this protein and a WT control were irradiated with UV-B light. Turbidity was measured at 0, 30, 45, and 60 min using the absorbance signal at 600 nm (Figure 4). Even after 60 min, γS-WT exhibited minimal aggregation, with an OD of 0.1 a.u. after 60 min. In contrast, γS-W163S had an OD of 0.8 a.u. after 45 min and 1 a.u. after 60 min, a factor of 10 higher than γS-WT. Bottom-up proteomics experiments on irradiated samples of both γS-WT and γS-W163S show numerous PTMs, including single oxidation of all four Trp residues and several Met and His residues, as well as double and triple oxidation of several Cys residues (Figure S3).

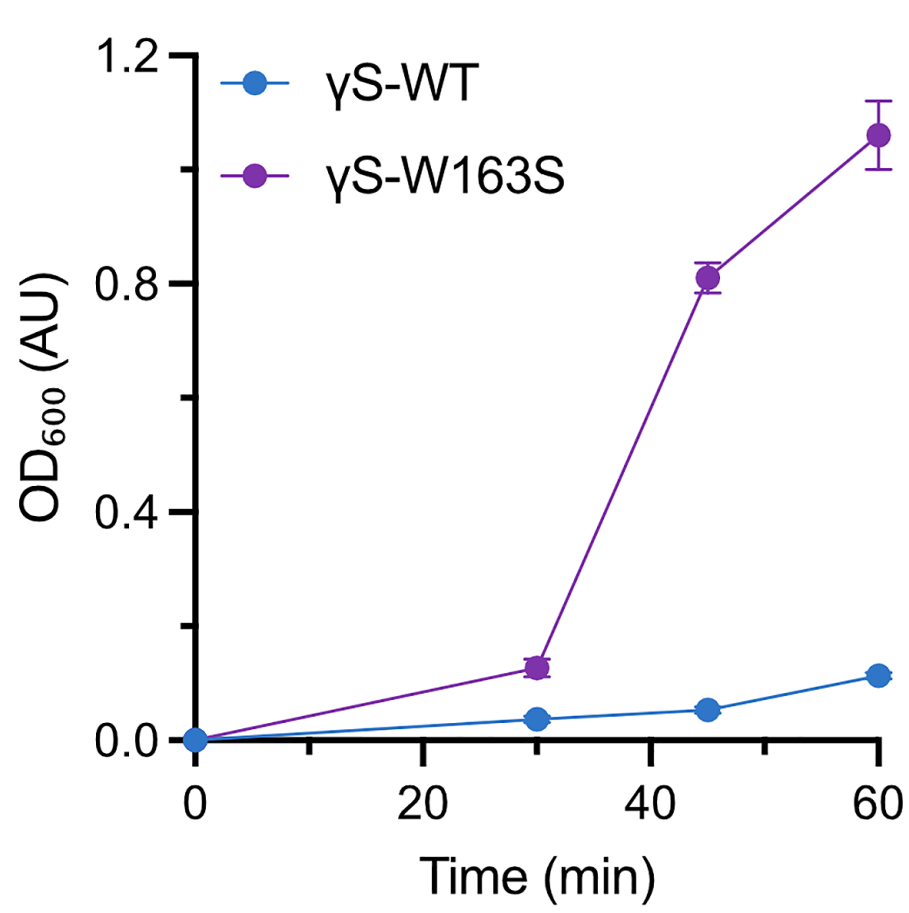

Figure 4. Turbidity measurement for γS-WT (blue) and γS-W163S (purple) as a function of UV-B irradiation time. Turbidity was monitored using the absorption at 600 nm. Error bars represent the standard deviation of three replicates.

To further assess the extent of structural perturbations to γS-W163S, two-dimensional ^{1}H-^{15}N HSQC spectra were collected (Figure 5A). Although the spectrum for γS-W163S (purple) is reasonably well resolved, significant differences in both the ^{1}H and ^{15}N chemical shifts are observed relative to γS-WT (blue). It is possible to assign the chemical shifts for most residues in the N-terminal domain and some in the C-terminal domain; however, many peaks corresponding to residues in the C-terminal domain cannot be identified by comparing these spectra in the absence of three-dimensional NMR experiments for full resonance assignment (Figure 5B). From these spectra, it is possible to say that the C-terminal domain of γS-W163S remains largely folded at room temperature, as indicated by the chemical-shift dispersion of the signals from the backbone amide protons, but is extensively structurally perturbed relative to γS-WT. If the C-terminal domain were unfolded, this spectrum would be characterized by much less chemical-shift dispersion, particularly in the ^{1}H dimension. Proton chemical shifts for disordered proteins tend to cluster narrowly in the region between 7.5 and 8.5 ppm. This suggests that, as with other structural crystallin variants, the hydrogen-bonding network of γS-W163S is rearranged to accommodate a major structural disruption without full unfolding.

γS-WT and γS-W163(5HTP) exhibit comparable internal motions globally

We previously hypothesized that the lowered onset temperature of thermal aggregation of γS upon W163

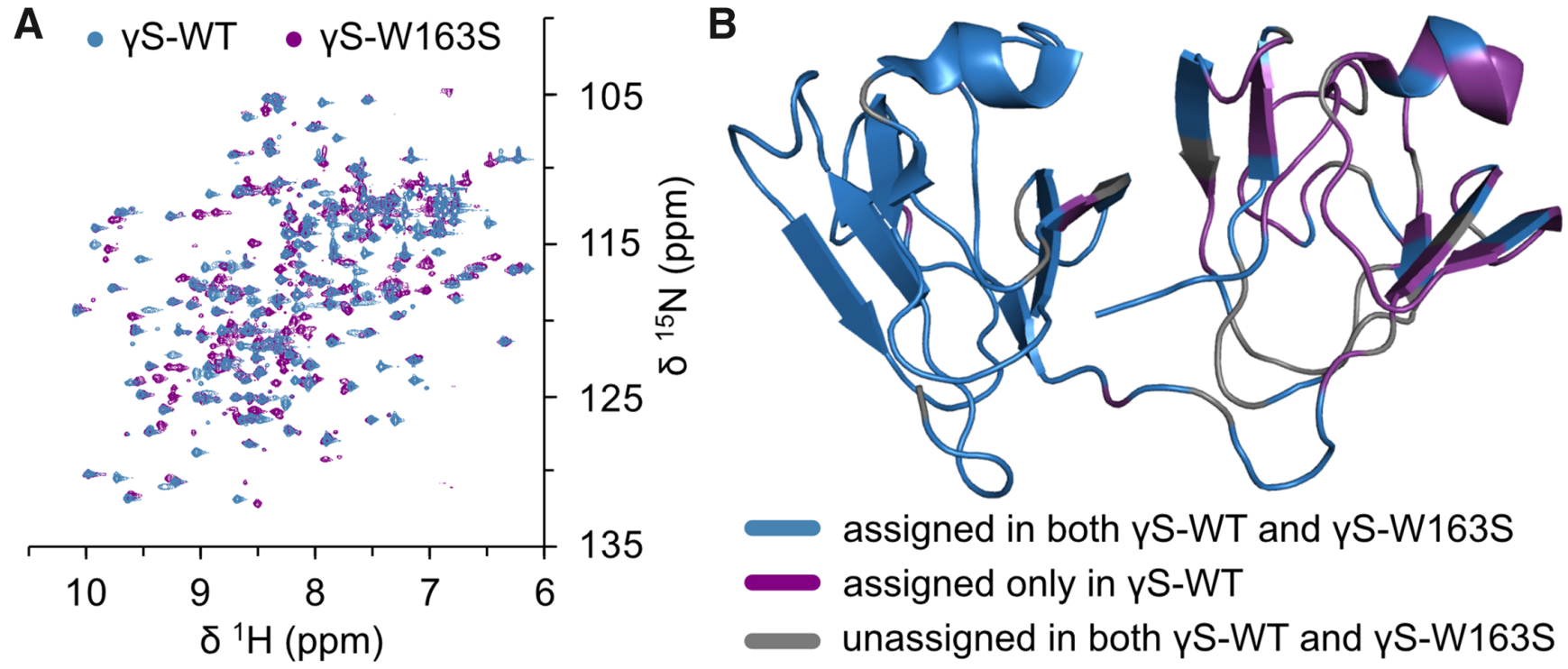

Figure 5. Conformational differences between γS-W163S and γS-WT are localized to the C-terminal domain. (A) Two-dimensional ^{1}H-^{15}N HSQC spectra both show chemical-shift dispersion consistent with a folded protein; however, many differences in chemical shifts are observed. (B) Most of the residues in the N-terminal domain of γS-W163S can be assigned by comparison to the spectrum of γS-WT, but many differences are observed in the C-terminal domain, indicating substantial conformational rearrangement.

oxidation arises from increased transient unfolding, which would be reflected in the relative internal motions of γS-WT and γS-W163(5HTP). To characterize these dynamics, we measured longitudinal (R_1) and transverse (R_2) relaxation rates and ^{1}H-^{15}N HetNOE (Figures 6A, 6B, S4, and S5) of these proteins in solution. Parameters calculated from these data are summarized in Table 1. The ratio of R_2 to R_1 is commonly used as a gauge of slower motions: higher values suggest conformational exchange contributions on the μs-to-ms timescale.[51,52] The average R_2/R_1 of γS-WT collected at 800 MHz is 22 ± 3, which is slightly lower than 25.6 reported by Forsythe et al.,[14] likely due to the elevated temperature in our experiments (25°C compared to 22°C). γS-W163(5HTP) shows a nearly identical average R_2/R_1 at 800 MHz (22 ± 3) as well as a slight increase relative to γS-WT that is within the margin of uncertainty at 500 MHz (γS-WT, 10.4 ± 0.7; γS-W163(5HTP), 10.6 ± 0.9). Such a trend is also observed in the average HetNOE values, which indicate that faster motions (on the

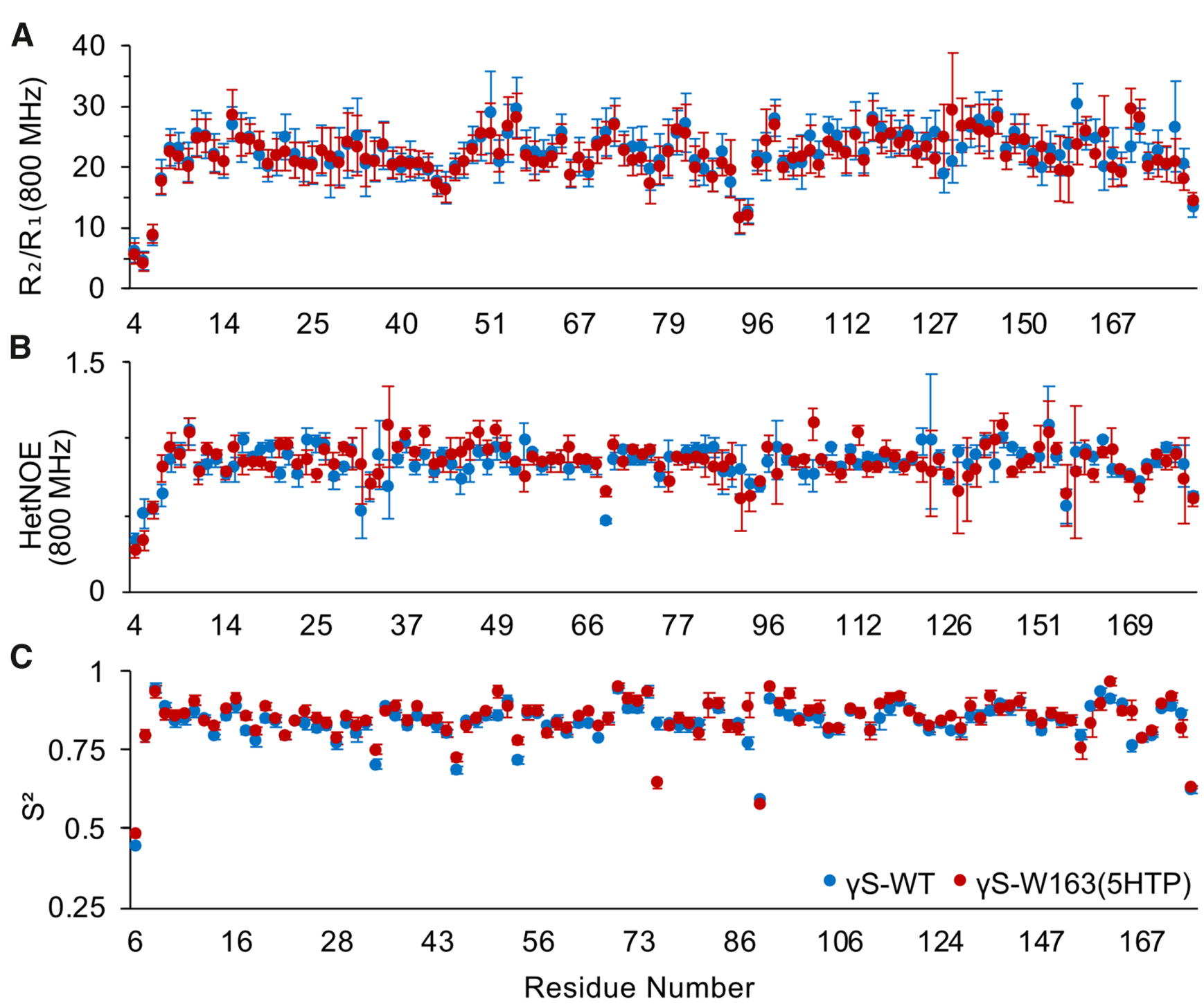

Figure 6. Representative ^{1}H-^{15}N relaxation data of γS-WT and γS-W163(5HTP) are plotted along the backbone of γS. Points indicate the calculated value, and solid error bars denote the fitting-derived (for R_2/R_1 and S^2) or intensity-derived (for HetNOE) uncertainty. Residues that were not detected, assigned, or fitted in each data set are excluded. (A) R_2/R_1 and (B) HetNOE data acquired at 800 MHz are shown. (C) S^2 values were estimated via Lipari-Szabo model-free analysis of the relaxation data collected at 500 and 800 MHz.

Table 1. Backbone dynamics parameters were calculated by fitting the ^{1}H-^{15}N NMR relaxation data and averaged, revealing negligible differences in internal motions between γS-WT and γS-W163(5HTP)

Parameter		γS-WT	γS-W163(5HTP)	*p*-value
R_2/R_1	(800 MHz)	22 ± 3	22 ± 3	0.0001
	(500 MHz)	10.4 ± 0.7	10.6 ± 0.9	<0.0001
HetNOE	(800 MHz)	0.83 ± 0.07	0.84 ± 0.08	0.59
	(500 MHz)	0.78 ± 0.04	0.82 ± 0.07	0.0001
S^2		0.84 ± 0.01	0.85 ± 0.02	0.0001
τ_m (ns)		11.56	11.56	–

S^2 and R_2/R_1 values are given as mean ± average fitting-derived uncertainty. ^{1}H-^{15}N heteronuclear NOE (HetNOE) values are given as mean ± average intensity-derived uncertainty. *p*-values were calculated using a two-tailed paired *t* test.

ps-to-ns timescale) also do not differ appreciably on a global level. The average HetNOE values at 800 MHz, 0.83 ± 0.07 for γS-WT and 0.84 ± 0.08 for γS-W163(5HTP), also closely match the mean value reported for γS-WT of 0.82.[14]

The average generalized order parameters (S^2) and overall rotational correlation times (τ_m) were estimated using the Lipari-Szabo model-free analysis of the relaxation data (Figures 6C and S6).[41,53] S^2 values report the degree of restriction of N–H-bond vector motion on fast (ps-to-ns) timescales. The nearly identical average S^2 values of γS-WT (0.84 ± 0.01) and γS-W163(5HTP) (0.85 ± 0.02) are consistent with the HetNOE analysis above; these estimates also agree with 0.86, a previously reported average for γS-WT.[14] The τ_m of γS-WT and γS-W163(5HTP) is 11.56 ns. This value is similar but not identical to those reported in the literature for γS-WT and a different variant (11.9 ns for γS-WT, 11.8 ns for γS-N14D,[14] and 11.82 ns for γS-G57W [11]), possibly because we employed an axially symmetric rotational diffusion tensor rather than an isotropic model. Nonetheless, γS-WT and γS-W163(5HTP) sharing the same τ_m value corroborates our previous findings that W163 oxidation does not affect the overall fold or the hydrodynamic size at ambient temperature.[27]

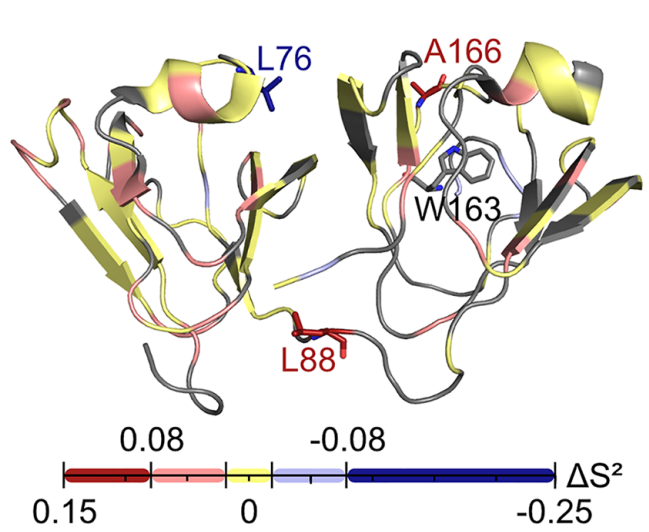

Figure 7. Residue-level changes in fast motions caused by W163 oxidation, quantified using ΔS^2 values between γS-WT and γS-W163(5HTP), counterbalance each other. The structure of γS (PDB: 2M3T)[54] is presented as following: residues with ΔS^2 values above 0.08 (dark red) or below −0.08 (dark blue) are shown in ball-and-stick representation. Residues with $|\Delta S^2|$ values smaller than 0.08 are colored pastel red (positive) and pastel blue (negative). Residues with ΔS^2 values within the average propagated margin of uncertainty are colored light yellow. Residues that were not detected, assigned, or fitted in either data set are shown in gray, including W163 (its indole side chain is shown in ball-and-stick representation).

Local perturbations in fast motions caused by W163 oxidation offset one another

The similarity between the average values and global parameters, however, masks a localized redistribution of dynamics. The solution NMR structure of γS (PDB: 2M3T)[54] was colored using the ΔS^2 between γS-WT and γS-W163(5HTP) for each residue (Figure 7). Although mild perturbations, slightly positive for the most part, are spotted throughout the protein, oxidation-induced changes in backbone flexibility surrounding W163 show notable patterns. A short segment of residues preceding W163, such as R158, K159, and D162, exhibits reduced S^2 in γS-W163(5HTP) relative to γS-WT ($\Delta = -0.042$, -0.053, and -0.035, respectively), indicating increased flexibility in the loop leading up to the short α-helix containing W163. In contrast, A166, located on the other side of the oxidation site, is considerably more rigid in γS-W163(5HTP) ($S^2 = 0.874$) compared to γS-WT ($S^2 = 0.763$; $\Delta = 0.111$). The same trend of increased rigidity is observed to a lesser degree for another residue, G164 ($\Delta = 0.054$). Furthermore, V170, whose relaxation data could not be fitted using the model-free analysis, exhibits a substantial increase in R_2/R_1 ($\Delta = 6$ at 800 MHz and 3 at 500 MHz) upon W163 oxidation, suggesting potential sampling of higher-energy states on slower timescales (μs-to-ms).

Aside from the residues surrounding W163, L76, located in a loop in the N-terminal domain, and L88, located in the linker connecting the two Greek key domains, also undergo counteracting perturbations. L76 exhibits increased fast motions in γS-W163(5HTP) compared to γS-WT ($\Delta S^2 = -0.19$), whereas L88 is rigidified in the presence of the oxidation mimic ($\Delta S^2 = 0.12$). These observations are less surprising given that other PTMs, such as deamidation, have also been found to shift the local conformational

dynamics of regions that are distant from the damage.[55] Collectively, these shifts in local dynamics caused by W163 oxidation likely have compensatory effects on global stability, which further rationalize the comparable unfolding trends between γS-WT and γS-W163(5HTP) under thermal stress (Figure 2) and chemical stress (Figure 3).[27] These results raise the possibility that long-lived proteins, such as crystallins, have evolved mechanisms for damage compensation by distributing structural changes rather than undergoing local unfolding: this is a hypothesis that bears further investigation.

DISCUSSION

Our characterization of γS-W163S aimed to further dissect the significance of W163 in γS-WT that renders it remarkably aggregation resistant. Although γS-W163S is similar in overall structure to γS-WT, the absence of a core hydrophobic residue in its C-terminal domain indeed destabilizes its overall fold. Its midpoint unfolding temperature and denaturant concentration are notably lower than those of γS-W163(5HTP), even farther from those of γS-WT. All three proteins show largely two-state unfolding under thermal and chemical stress (Figures 2A, 2B, and 3A–3C). The lack of intermediate states corroborates the well-established superior stability of the C-terminal domain compared to its N-terminal counterpart.[33,56] In both γS and γD, mutations in the N-terminal domain can generate partially unfolded intermediates on the path to fully unfolded states, unlike the alterations in the C-terminal end of γS mimicked in our variants.[22,57–59] The progressive reduction in stability between our γS variants—as a smaller, polar moiety is introduced, and then a larger, aromatic ring is removed from this site—does not lead to a concomitant increase in aggregation propensity. Instead, γS-W163S remains monomeric at physiological temperature alongside γS-WT, while γS-W163(5HTP) oligomerizes. A similar trend was previously reported for an engineered variant, γS-G106V, compared to its cataract-associated, symmetry-related counterpart, γS-G18V: although the former is substantially less stable, the latter is far more aggregation prone.[17] Another example of this stability-aggregation paradox is the S130P variant of γD-crystallin, which exhibits exceptional solubility despite its lack of conformational stability compared to the WT and a variant optimized for thermodynamic and kinetic stability, M69Q.[60]

A theme that has emerged from many studies of cataract-associated variants of βγ-crystallins is that mutations and PTMs that do not cause notable changes in the overall fold under ambient environments still have detrimental effects under stress conditions. Importantly, the resulting destabilization and aggregation are also decoupled from each other.[16,30,61,62] The mechanistic rationale for such behavior varies by protein, ranging from altered hydration shell dynamics,[8] increased hydrophobic surface area,[63–65] and perturbed intermolecular interaction networks[15,21] to disrupted domain interfaces.[22,66] The oxidized variant γS-W163(5HTP) reiterated this theme, aggregating well below physiological temperature despite only marginal differences in stability compared to γS-WT.[27]

These proteins, alongside the novel serine variant (γS-W163S) we introduce herein, undergo thermal aggregation, monitored using DLS, 20°C–30°C below their respective midpoint temperatures despite largely intact β-sheet structures detected via CD spectroscopy. We interpret this trend as indicating that larger oligomers, to the extent that they are observed in these experiments, contain native-like monomers without large-scale unfolding. This analysis is consistent with results reported for mutant γD types, including the cataract-related P23T variant[21] and several engineered variants where key Cys residues are replaced with Ser or Ala.[67] One factor that complicates the joint interpretation of CD and DLS data is varied sample concentrations: CD measurements were made at 0.25 mg mL^{-1}, whereas DLS correlograms were collected at 1 mg mL^{-1} under comparable temperature gradients. Based on prior results for crystallins as well as other proteins, we expect aggregation to have a strong concentration dependence, which was not investigated in this study.

Although γS-W163S was chosen primarily to systematically evaluate the role of W163 in the stability and solubility of γS, this missense variant is also reported in human genome data sets. As of yet, this mutation has unknown clinical significance, likely reflecting its extremely low allele frequency (0.000002%) and limited genotype representation.[45] Based on our results, we hypothesize that γS-W163S may have mild to moderate effects on crystallin aggregation *in vivo*; we predict that it may be correlated with early-onset (but not childhood) cataract, particularly if exacerbated by other risk factors such as high UV exposure or diabetes. If clinical consequences are observed in the future, these results may be helpful for understanding patient outcomes.

The removal of the W163 indole group in γS-W163S causes a substantial reduction in fluorescence quenching (manifesting as an increased fluorescence signal) much stronger than for the previously investigated W163F variant.[7] The strong enhancing effect of the W163S mutation on the fluorescence signal of γS-crystallin led us to develop a hypothesis that the W163S mutation would increase susceptibility to

UV-light damage at other sites by removing the indole chromophore of the C-terminal FRET acceptor. This hypothesis was supported by the dramatic increase in aggregation observed for γS-W163S compared to γS-WT upon UV-B irradiation (Figure 4). This suggests that UV-light damage even under ambient conditions may contribute to the increased aggregation propensity of γS-W163S.

Proteomic analysis of the soluble fraction after UV-B irradiation indicate substantial populations of peptides from both γS-W163S and γS-WT modified at all four Trp sites (Figure S3), in contrast to our previous study using hydroxyl radical production by γ-irradiation[23]: there, only the solvent-exposed W163 was modified. In γS-WT, our proteomics data indicate much more oxidation of W47 and W163 (the N-terminal and C-terminal FRET acceptors, respectively) compared to the donors, W73 and W137. However, these data must be interpreted cautiously because only the soluble fraction from each sample was analyzed—the observed preference for oxidation of the FRET acceptors may indicate instead that oxidation of the donors causes catastrophic aggregation, preventing these modifications from showing up in the proteomic data. Discriminating between these two scenarios will be a topic for future investigation.

Given these observations of the W163 oxidation mimic, we initially expected to observe an increase in internal motions around the site of oxidation in γS-W163(5HTP), particularly on the μs-to-ms scale, during which aggregation-competent conformations could be accessed. Instead, our results are inconsistent with the "transient unfolding" model of aggregation that is often proposed for aggregation-prone crystallins. This idea posits that aggregation in crystallin variants with only slightly reduced stability is primarily due to the protein transiently accessing highly aggregation-prone states that are never the most abundant species in solution but cause irreversible aggregation when are trapped by contact with another protein monomer. If this mechanism were very important to aggregation in γS-W163(5HTP), large increases in μs-to-ms around the site of the oxidative damage would be observed. The lack of altered dynamics on this timescale indicates that another mechanism is responsible for the higher aggregation propensity of γS-W163(5HTP).

A comparable study by other researchers characterized deamidation mimics of γS using NMR relaxation, reporting a near 4-fold increase in the number of residues exhibiting significant R_{ex} terms between γS-WT (10) and γS-N14D (37).[14] Based on this comparison, the authors concluded that the deamidation mimic samples a much more heterogeneous ensemble of conformations compared to γS-WT, some of which would serve as precursors for aggregation. Our model-free analysis of the backbone dynamics of γS-W163(5HTP), however, also listed ten residues with an R_{ex} term—comparable to γS-WT. Instead, we observed local differences in fast motions, on the ps-to-ns scale, between γS-WT and γS-W163(5HTP). The discrepancies surrounding W163 correspond well with the loss or gain of intramolecular H-bonds identified using thermal ^{1}H-^{15}N HSQC experiments reported in our prior study.[27] R158 exhibits increased flexibility in γS-W163(5HTP), perhaps because it is no longer constrained by an intramolecular H-bond, whereas A166 is more rigid in γS-W163(5HTP), possibly because it engages in one. Beyond the immediate site of oxidation, a few other residues also exhibit contrasting S_2 and R^2/R^1 values between γS-WT and γS-W163(5HTP). Taken together, our findings indicate that W163 oxidation locally redistributes intramolecular contacts to create a distinct set of tightly or loosely packed regions within a γS monomer. The subtle perturbations to the equilibrium between conformational substates of γS likely increase the probability of aberrant interactions between non-native surfaces of folded monomers. Furthermore, studying how these PTM mimics influence WT aggregation in heterogeneous mixtures would recapitulate the stochastic accumulation of covalent damage over time, as observed in the aging eye lens. This represents a promising direction for future study.

DATA AND CODE AVAILABILITY

- All data are available at Mendeley data (https://doi.org/10.17632/xjvy2vw839.3).
- All plasmids generated for this study have been deposited at Addgene (#255529 for γ-W163S and #255530 for γ-W163(5HTP)).
- No original code was generated for this study.
- Any additional information required to reanalyze the data reported in this paper is available from the corresponding author upon request.

ACKNOWLEDGMENTS

We acknowledge Dmitry Fishman of the UCI Laser Spectroscopy Labs, Ben Katz and Felix Grün of the UCI Mass Spectrometry Facility, and Suvrajit Sengupta of the UCI NMR Facility for access to instrumentation and assistance with data collection. We also acknowledge Zane Long at the UCI Ocular Mass Spectrometry, Lipidomics, and Proteomics Core for running samples on the QExactive mass spectrometer and assistance with data analysis. The authors acknowledge support from NIH grant P30 EY034070 to the Gavin Herbert Eye Institute at the University of California, Irvine. This work was supported by the National Institutes of Health under award number R21EYEY035792 to R.W.M., the UCI Undergraduate Research Opportunities Program under award number IRT005531, and the National Science Foundation under award number 2024276.

AUTHOR CONTRIBUTIONS

Y.S. and R.W.M. planned and designed the experiments; Y.S., A.A., M.I., T.K.D., L.X., M.K., and E.M.W. performed the experiments; and Y.S. and R.W.M. performed data analysis and prepared figures. The manuscript was written and edited by Y.S. and R.W.M., and all authors approved the final version of the manuscript.

DECLARATION OF INTERESTS

The authors declare no competing interests.

SUPPLEMENTAL INFORMATION

Supplemental information can be found online at https://doi.org/10.1016/j.bpr.2026.100291.

REFERENCES

1. Keskin, O., N. Tuncbag, and A. Gursoy. 2016. Predicting protein–protein interactions from the molecular to the proteome level. *Chem. Rev.* 116:4884–4909. https://doi.org/10.1021/acs.chemrev.5b00683.
2. Baldwin, A. J., T. P. J. Knowles, ..., C. M. Dobson. 2011. Metastability of native proteins and the phenomenon of amyloid formation. *J. Am. Chem. Soc.* 133:14160–14163. https://doi.org/10.1021/ja2017703.
3. Dill, K. A., and J. L. MacCallum. 2012. The protein-folding problem, 50 years on. *Science.* 338:1042–1046. https://doi.org/10.1126/science.1219021.
4. Zhao, H., P. H. Brown, ..., P. Schuck. 2011. The molecular refractive function of lens γ-crystallins. *J. Mol. Biol.* 411:680–699. https://doi.org/10.1016/j.jmb.2011.06.007.
5. Nooren, I. M. A., and J. M. Thornton. 2003. Diversity of protein–protein interactions. *EMBO J.* 22:3486–3492. https://doi.org/10.1093/emboj/cdg359.
6. Acosta-Sampson, L., and J. King. 2010. Partially folded aggregation intermediates of human γD-γC-and γS-crystallin are recognized and bound by human αB-crystallin chaperone. *J. Mol. Biol.* 401:134–152. https://doi.org/10.1016/j.jmb.2010.05.067.
7. Chen, J., P. R. Callis, and J. King. 2009. Mechanism of the very efficient quenching of tryptophan fluorescence in human γD-and γS-crystallins: The γ-crystallin fold may have evolved to protect tryptophan residues from ultraviolet photodamage. *Biochemistry.* 48:3708–3716. https://doi.org/10.1021/bi802177g.
8. Huang, K.-Y., C. N. Kingsley, ..., S. Han. 2016. Stability of protein-specific hydration shell on crowding. *J. Am. Chem. Soc.* 138:5392–5402. https://doi.org/10.1021/jacs.6b01989.
9. Ma, Z., G. Piszczek, ..., J. F. Hejtmancik. 2009. The G18V CRYGS mutation associated with human cataracts increases γS-crystallin sensitivity to thermal and chemical stress. *Biochemistry.* 48:7334–7341. https://doi.org/10.1021/bi900467a.
10. Vendra, V. P. R., S. Chandani, and D. Balasubramanian. 2012. The mutation V42M distorts the compact packing of the human γS-crystallin molecule, resulting in congenital cataract. *PLoS One.* 7:e51401. https://doi.org/10.1371/journal.pone.0051401.
11. Bari, K. J., S. Sharma, and K. V. R. Chary. 2019. Conformational dynamics study on human γS-crystallin as an efficient route to childhood blindness. *Biochem. Biophys. Res. Commun.* 511:679–684. https://doi.org/10.1016/j.bbrc.2019.02.124.
12. Takemoto, L., and C. M. Sorensen. 2008. Protein–protein interactions and lens transparency. *Exp. Eye Res.* 87:496–501. https://doi.org/10.1016/j.exer.2008.08.018.
13. Budnar, P., R. Tangirala, ..., C. M. Rao. 2022. Protein aggregation and cataract: Role of age-related modifications and mutations in α-crystallins. *Biochemistry.* 87:225–241. https://doi.org/10.1134/S000629792203004X.
14. Forsythe, H. M., C. J. Vetter, ..., K. J. Lampi. 2019. Altered protein dynamics and increased aggregation of human γS-crystallin due to cataract-associated deamidations. *Biochemistry.* 58:4112–4124. https://doi.org/10.1021/acs.biochem.9b00593.
15. Vetter, C. J., D. C. Thorn, ..., K. J. Lampi. 2020. Cumulative deamidations of the major lens protein γS-crystallin increase its aggregation during unfolding and oxidation. *Protein Sci.* 29:1945–1963. https://doi.org/10.1002/pro.3915.
16. Norton-Baker, B., P. Mehrabi, ..., R. W. Martin. 2022. Deamidation of the human eye lens protein γS-crystallin accelerates oxidative aging. *Structure.* 30:763–776.e4. https://doi.org/10.1016/j.str.2022.03.002.
17. Roskamp, K. W., C. N. Paulson, ..., R. W. Martin. 2020. Function and aggregation in structural eye lens crystallins. *Acc. Chem. Res.* 53:863–874. https://doi.org/10.1021/acs.accounts.0c00014.
18. Zhao, H., M. T. Magone, and P. Schuck. 2011. The role of macromolecular crowding in the evolution of lens crystallins with high molecular refractive index. *Phys. Biol.* 8:046004. https://doi.org/10.1088/1478-3975/8/4/046004.
19. Henzler-Wildman, K., and D. Kern. 2007. Dynamic personalities of proteins. *Nature.* 450:964–972. https://doi.org/10.1038/nature06522.
20. Chiti, F., and C. M. Dobson. 2017. Protein misfolding, amyloid formation, and human disease: a summary of progress over the last decade. *Annu. Rev. Biochem.* 86:27–68. https://doi.org/10.1146/annurev-biochem-061516-045115.
21. Boatz, J. C., M. J. Whitley, ..., P. C. A. van der Wel. 2017. Cataract-associated P23T γD-crystallin retains a native-like fold in amorphous-looking aggregates formed at physiological pH. *Nat. Commun.* 8:15137. https://doi.org/10.1038/ncomms15137.
22. Volz, S., J. R. Malone, ..., S. Marqusee. 2025. Cataract-prone variants of γD-crystallin populate a conformation with a partially unfolded N-terminal domain under native conditions. *Proc. Natl. Acad. Sci. USA.* 122:e2410860122. https://doi.org/10.1073/pnas.2410860122.
23. Norton-Baker, B., M. A. Rocha, ..., R. W. Martin. 2022. Human γS-crystallin resists unfolding despite extensive chemical modification from exposure to ionizing radiation. *J. Phys. Chem. B.* 126:679–690. https://doi.org/10.1021/acs.jpcb.1c08157.
24. Asomugha, C. O., R. Gupta, and O. P. Srivastava. 2010. Identification of crystallin modifications in the human lens cortex and nucleus using laser capture microdissection and CyDye labeling. *Mol. Vis.* 16:476–494.
25. Kosinski-Collins, M. S., S. L. Flaugh, and J. King. 2004. Probing folding and fluorescence quenching in human γD crystallin Greek key domains using triple tryptophan mutant proteins. *Protein Sci.* 13:2223–2235. https://doi.org/10.1110/ps.04627004.
26. Xu, J., J. Chen, ..., J. R. Knutson. 2009. Femtosecond fluorescence spectra of tryptophan in human γ-crystallin mutants: Site-dependent ultrafast quenching. *J. Am. Chem. Soc.* 131:16751–16757. https://doi.org/10.1021/ja904857t.
27. Seo, Y., Z. G. Long, ..., R. W. Martin. 2026. Mimicking oxidative damage in γS-crystallin with site-specific incorporation of 5-hydroxytryptophan. *Biophys. Rep.* 6:100251. https://doi.org/10.1016/j.bpr.2026.100251.
28. Michiel, M., E. Duprat, ..., S. Finet. 2010. Aggregation of deamidated human βB2-crystallin and incomplete rescue by α-crystallin chaperone. *Exp. Eye Res.* 90:688–698. https://doi.org/10.1016/j.exer.2010.02.007.

29. Evans, P., C. Slingsby, and B. A. Wallace. 2008. Association of partially folded lens βB2-crystallins with the α-crystallin molecular chaperone. *Biochem. J.* 409:691–699. https://doi.org/10.1042/BJ20070993.

30. Brubaker, W. D., J. A. Freites, …, R. W. Martin. 2011. Separating instability from aggregation propensity in γS-crystallin variants. *Biophys. J.* 100:498–506. https://doi.org/10.1016/j.bpj.2010.12.3691.

31. Ficaretta, E. D., C. J. J. Wrobel, …, A. Chatterjee. 2022. A robust platform for unnatural amino acid mutagenesis in *E. coli* using the bacterial tryptophanyl-tRNA synthetase/tRNA pair. *J. Mol. Biol.* 434:167304. https://doi.org/10.1016/j.jmb.2021.167304.

32. Brubaker, W. D., and R. W. Martin. 2012. 1H, 13C, and 15N assignments of wild-type human γS-crystallin and its cataract-related variant γS-G18V. *Biomol. NMR Assign.* 6:63–67. https://doi.org/10.1007/s12104-011-9326-1.

33. Mills-Henry, I. A., S. L. Thol, …, J. A. King. 2019. Kinetic stability of long-lived human lens γ-crystallins and their isolated double Greek key domains. *Biophys. J.* 117:269–280. https://doi.org/10.1016/j.bpj.2019.06.006.

34. Davis, S. M., B. L. Romig, …, N. M. Loening. 2025. An improved variant of tobacco etch virus (TEV) protease that does not need reducing agents. *Protein Sci.* 34:e70049. https://doi.org/10.1002/pro.70049.

35. Micsonai, A., F. Wien, …, J. Kardos. 2015. Accurate secondary structure prediction and fold recognition for circular dichroism spectroscopy. *Proc. Natl. Acad. Sci. USA.* 112:E3095–E3103. https://doi.org/10.1073/pnas.1500851112.

36. Sprague-Piercy, M. A., E. Wong, …, R. W. Martin. 2020. Human αB-crystallin discriminates between aggregation-prone and function-preserving variants of a client protein. *Biochim. Biophys. Acta Gen. Subj.* 1864:129502. https://doi.org/10.1016/j.bbagen.2019.129502.

37. Cumberland, W. N., Y. Fong, …, S. De Rosa. 2015. Nonlinear calibration model choice between the four and five-parameter logistic models. *J. Biopharm. Stat.* 25:972–983. https://doi.org/10.1080/10543406.2014.920345.

38. Greene, R. F., and C. N. Pace. 1974. Urea and guanidine hydrochloride denaturation of ribonuclease, lysozyme, α-chymotrypsin, and β-lactoglobulin. *J. Biol. Chem.* 249:5388–5393.

39. Rafi, A., A. Sambreena, …, P. Ramesh. 2026. M2Viz: A tool for visualizing genetic or proteomic modifications and variants. *BMC Bioinf.* 27:143. https://doi.org/10.1186/s12859-026-06484-2.

40. Skinner, S. P., R. H. Fogh, …, G. W. Vuister. 2016. CcpNmr AnalysisAssign: a flexible platform for integrated NMR analysis. *J. Biomol. NMR.* 66:111–124. https://doi.org/10.1007/s10858-016-0060-y.

41. Cole, R., and J. P. Loria. 2003. Fast-modelfree: a program for rapid automated analysis of solution NMR spin-relaxation data. *J. Biomol. NMR.* 26:203–213. https://doi.org/10.1023/a:1023808801134.

42. Maciejewski, M. W., A. D. Schuyler, …, J. C. Hoch. 2017. NMRbox: a resource for biomolecular NMR computation. *Biophys. J.* 112:1529–1534. https://doi.org/10.1016/j.bpj.2017.03.011.

43. Kubiak, K., M. Kowalska, and W. Nowak. 2003. Molecular dynamics study of early events during photooxidation of eye lens protein γB-crystallin. *J. Mol. Struct. THEOCHEM.* 630:315–325. https://doi.org/10.1016/s0166-1280(03)00161-1.

44. Xia, Z., Z. Yang, …, R. Zhou. 2013. UV-radiation induced disruption of dry-cavities in human γD-crystallin results in decreased stability and faster unfolding. *Sci. Rep.* 3:1560. https://doi.org/10.1038/srep01560.

45. All of Us Research Program Investigators. 2024. All of Us research program data browser: CRYGS variant page. All of Us Research Hub [Online]. https://databrowser.researchallofus.org/variants/crygs.

46. Greenfield, N. J. 2006. Using circular dichroism spectra to estimate protein secondary structure. *Nat. Protoc.* 1:2876–2890. https://doi.org/10.1038/nprot.2006.202.

47. Mohr, B. G., C. M. Dobson, …, M. Muthukumar. 2013. Electrostatic origin of in vitro aggregation of human γ-crystallin. *J. Chem. Phys.* 139:121914. https://doi.org/10.1063/1.4816367.

48. Callis, P. R., and T. Liu. 2004. Quantitative prediction of fluorescence quantum yields for tryptophan in proteins. *J. Phys. Chem. B.* 108:4248–4259. https://doi.org/10.1021/jp0310551.

49. Makhatadze, G. I. 1999. Thermodynamics of protein interactions with urea and guanidinium hydrochloride. *J. Phys. Chem. B.* 103:4781–4785. https://doi.org/10.1021/jp990413q.

50. Pace, C. N., S. Treviño, …, J. M. Scholtz. 2004. Protein structure, stability and solubility in water and other solvents. *Philos. Trans. R. Soc. Lond. B Biol. Sci.* 359:1225–1235. https://doi.org/10.1098/rstb.2004.1500.

51. Palmer, A. G., 3rd 2001. NMR probes of molecular dynamics: overview and comparison with other techniques. *Annu. Rev. Biophys. Biomol. Struct.* 30:129–155. https://doi.org/10.1146/annurev.biophys.30.1.129.

52. Dyson, H. J. 2021. Early strides in NMR dynamics measurements. *Biochemistry.* 60:3452–3454. https://doi.org/10.1021/acs.biochem.1c00141.

53. Lipari, G., and A. Szabo. 1982. Model-free approach to the interpretation of nuclear magnetic resonance relaxation in macromolecules. 1. Theory and range of validity. *J. Am. Chem. Soc.* 104:4546–4559. https://doi.org/10.1021/ja00381a009.

54. Kingsley, C. N., W. D. Brubaker, …, R. W. Martin. 2013. Preferential and specific binding of human αB-crystallin to a cataract-related variant of γS-crystallin. *Structure.* 21:2221–2227. https://doi.org/10.1016/j.str.2013.09.017.

55. Takata, T., J. P. Smith, …, K. J. Lampi. 2010. Solvent accessibility of βB2-crystallin and local structural changes due to deamidation at the dimer interface. *Exp. Eye Res.* 91:336–346. https://doi.org/10.1016/j.exer.2010.05.019.

56. Mills, I. A., S. L. Flaugh, …, J. A. King. 2007. Folding and stability of the isolated Greek key domains of the long-lived human lens proteins γD-crystallin and γS-crystallin. *Protein Sci.* 16:2427–2444. https://doi.org/10.1110/ps.072970207.

57. Lee, S., B. Mahler, …, Z. Wu. 2010. A single destabilizing mutation (F9S) promotes concerted unfolding of an entire globular domain in γS-crystallin. *J. Mol. Biol.* 399:320–330. https://doi.org/10.1016/j.jmb.2010.04.003.

58. Ji, F., J. Jung, …, A. M. Gronenborn. 2013. The human W42R γD-crystallin mutant structure provides a link between congenital and age-related cataracts. *J. Biol. Chem.* 288:99–109. https://doi.org/10.1074/jbc.M112.416354.

59. Bari, K. J., S. Sharma, and K. V. R. Chary. 2018. Structural and functional characterization of a missense mutant of human γS-crystallin associated with dominant infantile cataracts. *Biochem. Biophys. Res. Commun.* 506:862–867. https://doi.org/10.1016/j.bbrc.2018.10.187.

60. Sahin, E., J. L. Jordan, …, C. J. Roberts. 2011. Computational design and biophysical characterization of aggregation-resistant point mutations for γD crystallin illustrate a balance of conformational stability and intrinsic aggregation propensity. *Biochemistry.* 50:628–639. https://doi.org/10.1021/bi100978r.

61. Serebryany, E., and J. A. King. 2014. The βγ-crystallins: native state stability and pathways to aggregation. *Prog. Biophys. Mol. Biol.* 115:32–41. https://doi.org/10.1016/j.pbiomolbio.2014.05.002.

62. Xu, W., J. Chen, …, X. Chen. 2024. A novel cataract-related mutation R10P in γA-crystallin increases susceptibility to thermal shock and ultraviolet radiation of γA-crystallin. *Biochem.*

Biophys. Res. Commun. 739:150585. https://doi.org/10.1016/j.bbrc.2024.150585.

63. Talla, V., C. Narayanan, …, D. Balasubramanian. 2006. Mutation causing self-aggregation in human γC-crystallin leading to congenital cataract. *Investig. Ophthalmol. Vis. Sci.* 47:5212–5217. https://doi.org/10.1167/iovs.06-0427.

64. Ji, F., L. M. I. Koharudin, …, A. M. Gronenborn. 2013. Crystal structure of the cataract-causing P23T γD-crystallin mutant. Proteins - Structure. *Proteins.* 81:1493–1498. https://doi.org/10.1002/prot.24321.

65. Indu, and R. Mishra. 2026. pH-driven distinct aggregation pathways of human γD-crystallin. *J. Phys. Chem. B.* 130:4060–4069. https://doi.org/10.1021/acs.jpcb.5c06855.

66. Hsueh, S.-S., S. S. S. Wang, …, T.-H. Lin. 2022. Insights to human γD-crystallin unfolding by NMR spectroscopy and molecular dynamics simulations. *Int. J. Mol. Sci.* 23:1591. https://doi.org/10.3390/ijms23031591.

67. Pu, Y., V. Seecharan, …, E. Serebryany. 2026. Dimerization of natively folded molecules drives misfolding and aggregation in Cys-depleted variants of cataract-associated human lens γD-crystallin. *J. Biol. Chem.* 302:113241. https://doi.org/10.1016/j.jbc.2026.113241.

Biophysical Reports, Volume *6*

Supplemental information

Tryptophan oxidation-induced dynamics highlight why stable γS-crystallin variants aggregate

Yeonseong Seo, Amanda Abiad, Micco Iwase, Tsoler K. Demerdjian, Linyuting Xing, Minseo Kim, Elizabeth M. Wingen, and Rachel W. Martin

Supplemental information for: Tryptophan oxidation-induced dynamics highlight why stable γS-crystallin variants aggregate

Yeonseong Seo[1], Amanda Abiad[1], Micco Iwase[2], Tsoler K. Demerdjian[3], Linyuting Xing[1], Minseo Kim[1], and Rachel W. Martin[1,4,*]

[1]Department of Chemistry, University of California, Irvine, Irvine, CA 92697, USA
[2]School of Biological Sciences, University of California, Irvine, Irvine, CA 92697, USA
[3]School of Population & Public Health, University of California, Irvine, Irvine, CA 92697, USA
[4]Department of Molecular Biology and Biochemistry, University of California, Irvine, Irvine, CA 92697, USA
[*]Correspondence: rwmartin@uci.edu

The following items are available for download online at DOI: 10.17632/xjvy2vw839.1: 1) fluorescence, circular dichroism (CD), and dynamic light scattering (DLS) characterization data for γS-WT, γS-W163(5HTP), and γS-W163S and 2) ^{1}H–^{15}N nuclear magnetic resonance (NMR) relaxation data for γS-WT and γS-W163(5HTP).

Figure S1: γS-W163S sequence was designed with 5′ and 3′ overhangs for Gibson assembly.

```
TGTTTAACTTTAAGAAGGAGATATACCATGGGACATCACCATCACCATCACGAAAATCTTTATTTTCAAGGCAGCAAAACG
GGCACCAAAATCACGTTCTACGAAGACAAAAATTTCCAAGGTCGCCGTTACGACTGTGACTGCGACTGTGCGGACTTCCAC
ACATACCTGTCCCGTTGCAACTCTATCAAAGTTGAAGGCGGCACCTGGGCTGTATACGAACGCCCGAACTTCGCTGGCTAC
ATGTACATCCTTCCGCAGGGCGAATACCCGGAATACCAGCGCTGGATGGGTCTGAACGACCGTCTGTCGTCTTGCCGCGCT
GTACATCTGCCGTCCGGCGGTCAGTACAAAATCCAGATCTTCGAAAAAGGCGACTTCTCCGGCCAGATGTACGAAACCACC
GAAGACTGCCCGTCTATCATGGAACAATTCCACATGCGCGAAATCCACTCTTGTAAAGTTCTGGAAGGCGTATGGATCTTC
TACGAACTGCCGAACTACCGCGGTCGCCAGTACCTGCTGGACAAAAAAGAATACCGCAAACCGATCGACAGCGGCGCGGCC
TCTCCGGCTGTACAGAGCTTCCGTCGTATCGTTGAATAATAACTCGAGCACCACCACCACCACCACTGAGATC
```

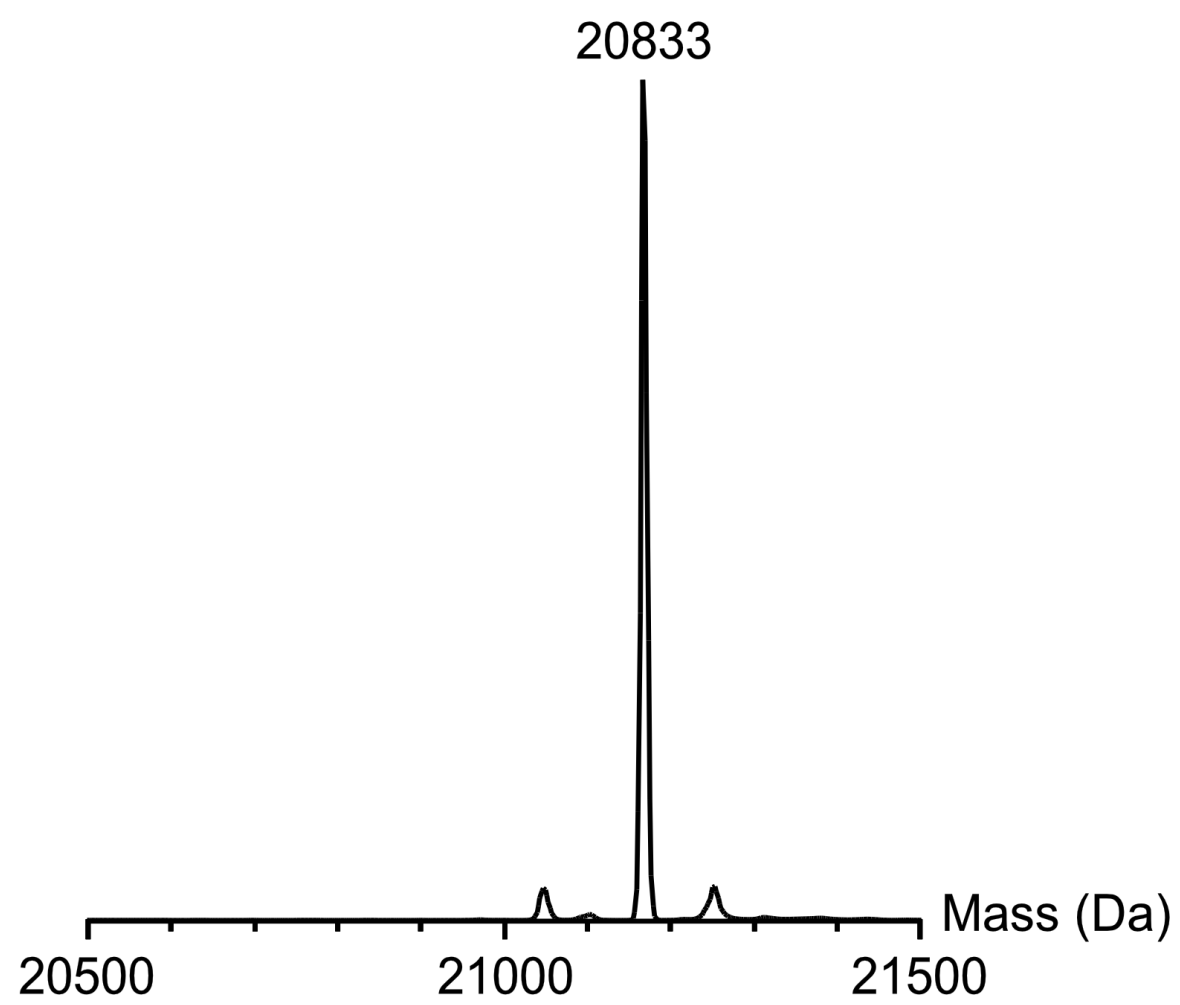

Figure S2: The reconstructed mass spectrum of γS-W163S showed a peak that corresponds to the expected mass calculated from its amino acid sequence.

Table 1: Protein secondary structure analysis of circular dichroism (CD) spectra of γS-WT, γS-W163(5HTP), and γS-W163S suggests a small increase in β-strand content as residue 163 is altered.[1]

**Structure**	**γS-WT**	**γS-W163(5HTP)**	**γS-W163S**
Helix	0.0%	0.0%	0.0%
Strand	49.5%	53.1%	55.7%
Others	50.5%	46.9 %	44.3%

Table 2: Destabilization of γS-W163S was quantified from thermal and chemical unfolding curves and compared to previously published values for γS-WT and γS-W163(5HTP) with permission (license number 6295410703193).[2] Parameters are given as mean $\pm$ standard deviation. Adjusted p-values were calculated using one-way ANOVA with Dunnett's multiple comparison test versus γS-WT.

**Parameter**	**γS-WT**	**γS-W163(5HTP)**	**p-value**	**γS-W163S**	**p-value**
T_m (°C)	78.1$\pm$0.4	72.8 $\pm$0.3	$<$0.0001	66.7 $\pm$0.5	$<$0.0001
Midpoint (M)	2.77 $\pm$0.08	2.47 $\pm$0.02	0.0005	1.73 $\pm$0.03	$<$0.0001
ΔG_{app} (kJ mol^{-1})	6.01 $\pm$0.03	5.5 $\pm$0.8	0.59	9.5 $\pm$0.8	0.0011
m (kJ/mol/M)	$-$0.75 $\pm$0.03	$-$0.7 $\pm$0.1	0.97	$-$1.4 $\pm$0.1	0.0005

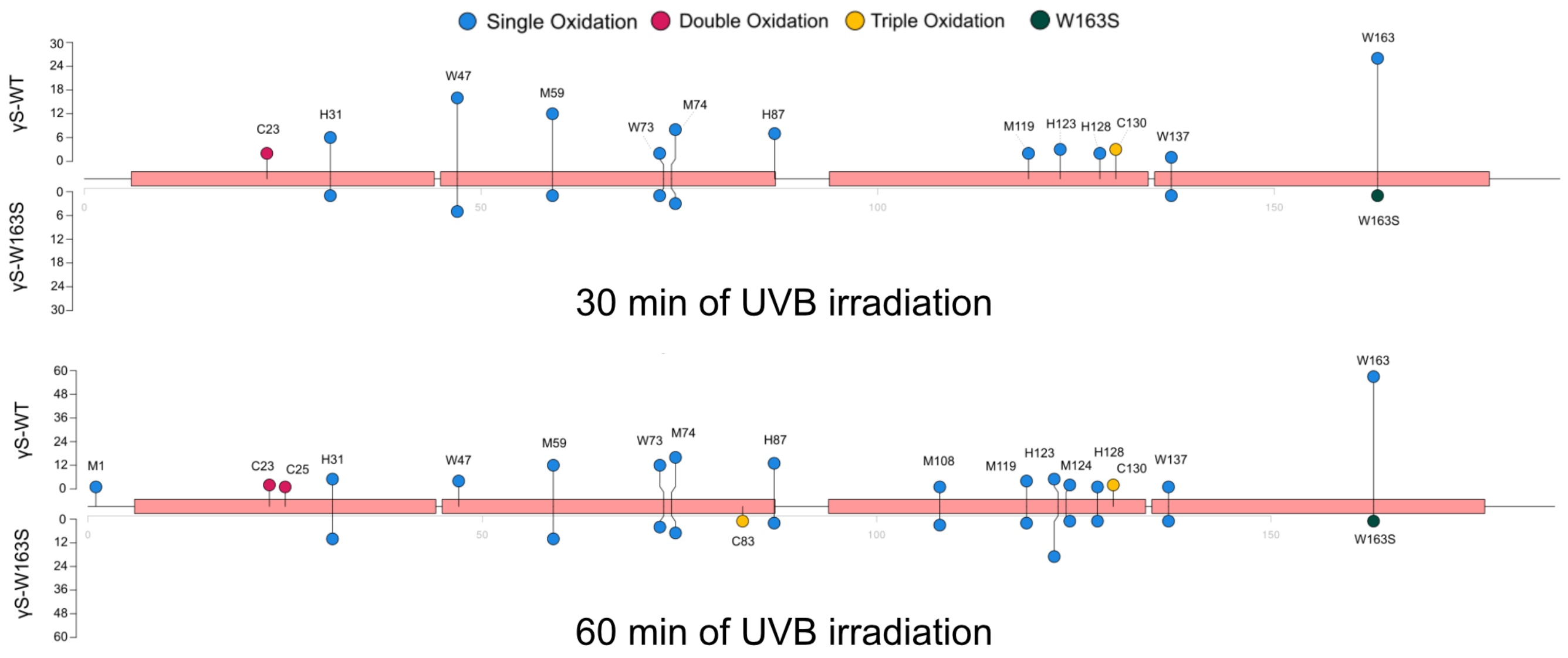

Figure S3: Lollipop plots showing modification sites for γS-WT (up) and γS-W163S (down). The top panel shows modifications after 30 minutes of UV-B irradiation, whereas the bottom panel shows modifications after 60 minutes. Single oxidations are shown in blue, double in red, and triple in yellow. Only the soluble fraction of the protein was digested here: modification sites on insoluble aggregates are therefore not observed.

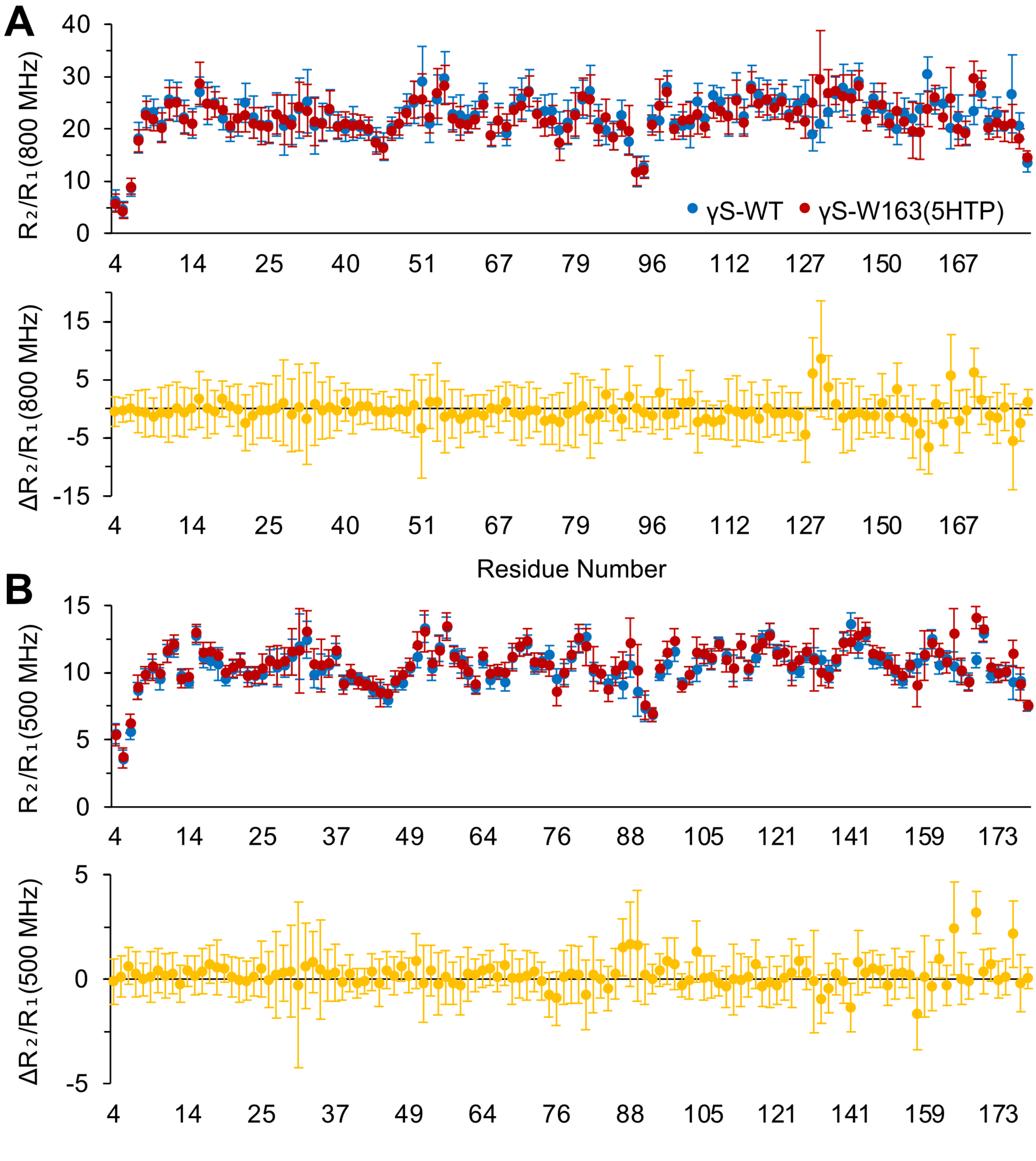

Figure S4: R_2/R_1 of γS-WT and γS-W163(5HTP) were calculated by fitting the ^{1}H–^{15}N NMR relaxation data collected at (A) 800 MHz and (B) 500 MHz. $\Delta R_2/R_1$ are plotted to show perturbations caused by W163 oxidation. Points indicate the calculated value and solid error bars denote the fitting-derived uncertainty (for R_2/R_1) and the propagated uncertainty (for $\Delta R_2/R_1$). Residues that were not detected, assigned, or fitted in either dataset are excluded.

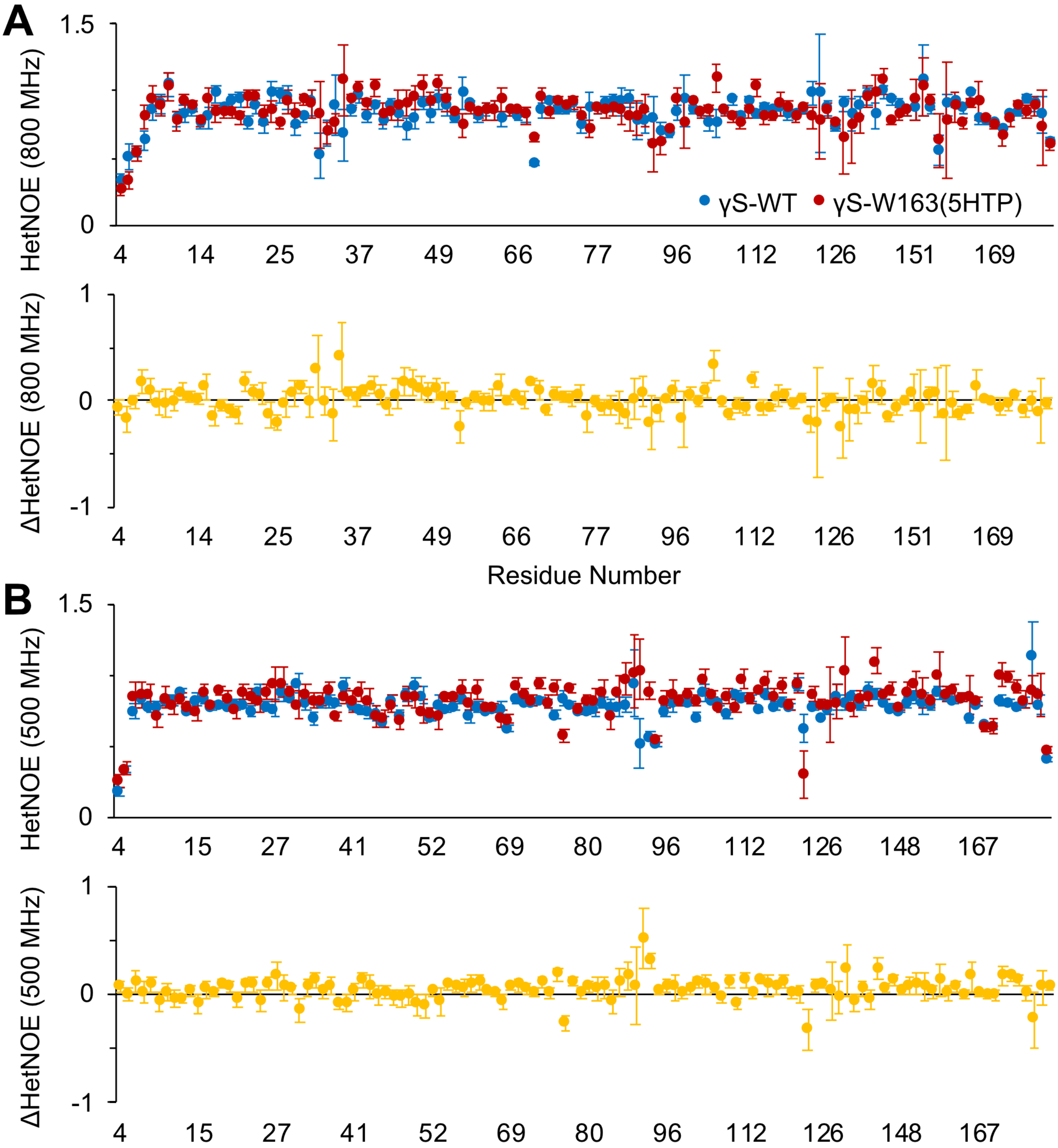

Figure S5: HetNOE of γS-WT and γS-W163(5HTP) were measured at (A) 800 MHz and (B) 500 MHz. ΔHetNOE are plotted to show perturbations caused by W163 oxidation. Points indicate the calculated value and solid error bars denote the intensity-derived uncertainty (for HetNOE) and the propagated uncertainty (for ΔHetNOE). Residues that were not detected, assigned, or fitted in either dataset are excluded.

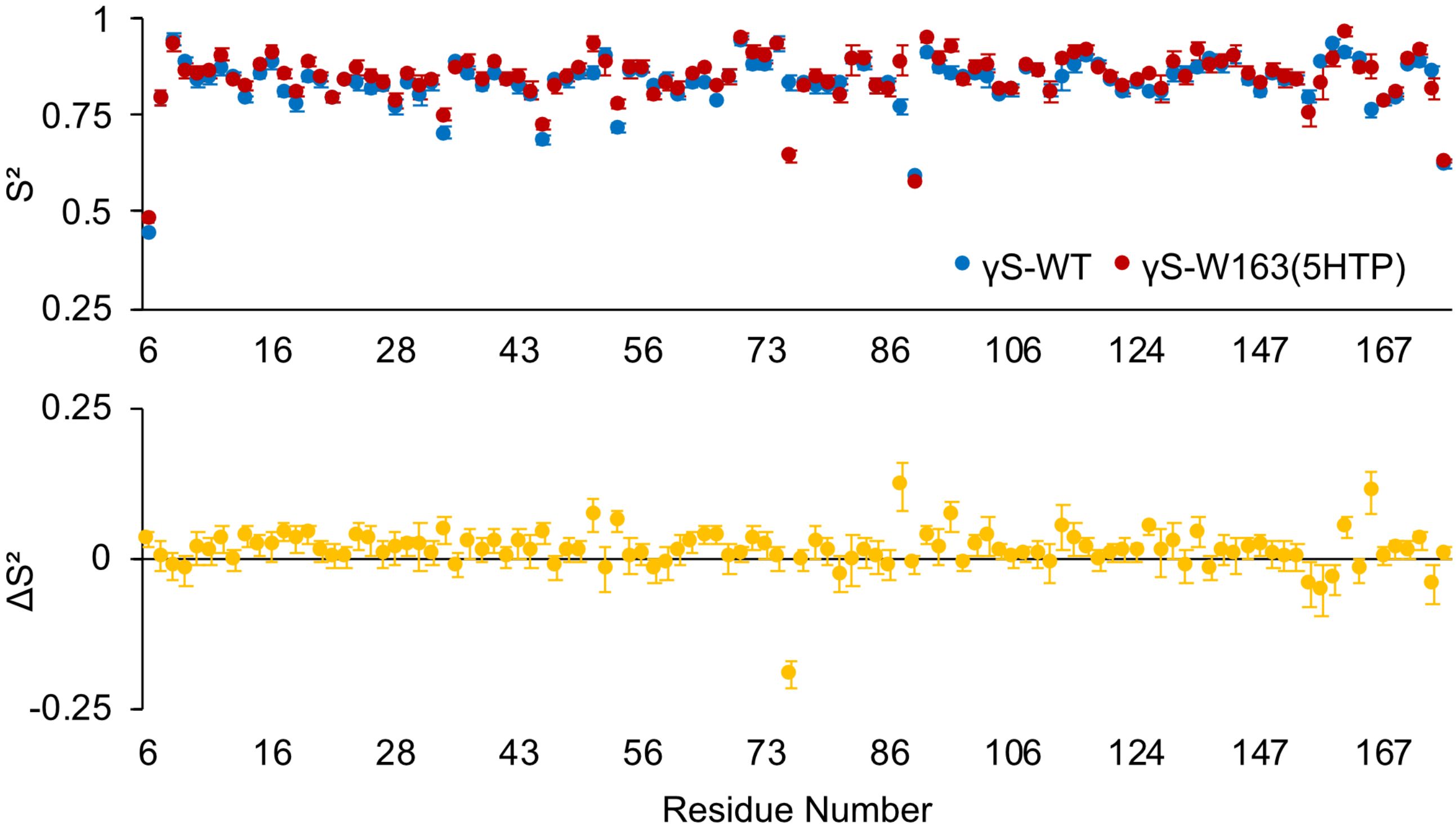

Figure S6: S^2 of γS-WT and γS-W163(5HTP) were estimated via Lipari–Szabo model-free analysis of the ^{1}H–^{15}N relaxation data. ΔS^2 are plotted to show perturbations caused by W163 oxidation. Points indicate the calculated value and solid error bars denote the fitting-derived uncertainty (for S^2) and the propagated uncertainty (for ΔS^2) of each residue. Residues that were not detected, assigned, or fitted in either dataset were excluded.

References

1. Micsonai, A., Wien, F., Kernya, L., Lee, Y.H., Goto, Y., Réfrégiers, M., and Kardos, J. (2015). Accurate secondary structure prediction and fold recognition for circular dichroism spectroscopy. Proceedings of the National Academy of Sciences *112*, E3095–E3103.

2. Seo, Y., Long, Z.G., Demerdjian, T.K., Avenido, A.A., Butts, C.T., and Martin, R.W. (2026). Mimicking oxidative damage in γS-crystallin with site-specific incorporation of 5-hydroxytryptophan. Biophysical Reports *6*, 100251.
